# Ovule identity mediated by pre-mRNA processing in Arabidopsis

**DOI:** 10.1371/journal.pgen.1007182

**Published:** 2018-01-12

**Authors:** Encarnación Rodríguez-Cazorla, Samanta Ortuño-Miquel, Héctor Candela, Lindsay J. Bailey-Steinitz, Martin F. Yanofsky, Antonio Martínez-Laborda, Juan-José Ripoll, Antonio Vera

**Affiliations:** 1 Área de Genética, Universidad Miguel Hernández, Campus de Sant Joan d’Alacant, Sant Joan d’Alacant, Alicante, Spain; 2 Instituto de Bioingeniería, Universidad Miguel Hernández, Campus de Elche, Elche, Alicante, Spain; 3 Division of Biological Sciences, Section of Cell and Developmental Biology, University of California San Diego, La Jolla, California, United States of America; University of California Berkeley, UNITED STATES

## Abstract

Ovules are fundamental for plant reproduction and crop yield as they are the precursors of seeds. Therefore, ovule specification is a critical developmental program. In *Arabidopsis thaliana*, ovule identity is redundantly conferred by the homeotic D-class genes *SHATTERPROOF1* (*SHP1*), *SHP2* and *SEEDSTICK* (*STK*), phylogenetically related to the MADS-domain regulatory gene *AGAMOUS* (*AG*), essential in floral organ specification. Previous studies have shown that the *HUA-PEP* activity, comprised of a suite of RNA-binding protein (RBP) encoding genes, regulates *AG* pre-mRNA processing and thus flower patterning and organ identity. Here, we report that the *HUA-PEP* activity additionally governs ovule morphogenesis. Accordingly, in severe *hua-pep* backgrounds ovules transform into flower organ-like structures. These homeotic transformations are most likely due to the dramatic reduction in *SHP1*, *SHP2* and *STK* activity. Our molecular and genome-wide profiling strategies revealed the accumulation of prematurely terminated transcripts of D-class genes in *hua-pep* mutants and reduced amounts of their respective functional messengers, which points to pre-mRNA processing misregulation as the origin of the ovule developmental defects in such backgrounds. RNA processing and transcription are coordinated by the RNA polymerase II (RNAPII) carboxyl-terminal domain (CTD). Our results show that *HUA-PEP* activity members can interact with the CTD regulator C-TERMINAL DOMAIN PHOSPHATASE-LIKE1 (CPL1), supporting a co-transcriptional mode of action for the *HUA-PEP* activity. Our findings expand the portfolio of reproductive developmental programs in which *HUA-PEP* activity participates, and further substantiates the importance of RNA regulatory mechanisms (pre-mRNA co-transcriptional regulation) for correct gene expression during plant morphogenesis.

## Author summary

Plant ovules are crucial reproductive structures in which the female gametophyte develops, giving rise to seeds after fertilization. Global food supply depends mainly on seed production, thus understanding the underlying regulatory mechanisms that orchestrate ovule development is vitally important. The establishment of ovule identity is a key process that largely relies on the MADS-box transcription factors that define the floral D-function. In Arabidopsis the D-class is represented by *SHP1*, *SHP2* and *STK*, the closest paralogs of *AG*, the master regulator of floral morphogenesis. Previous studies indicated that the post-transcriptional regulatory module termed “*HUA-PEP* gene activity” facilitates *AG* pre-mRNA maturation to secure *AG* function. Here we show that the *HUA-PEP* activity also targets D-class gene expression for the correct specification of ovule identity. Homeotic transformations of ovules into flower organ-like structures occur in plants in which the *HUA-PEP* function is compromised. In such backgrounds, prematurely terminated transcripts of *SHP1*, *SHP2* and *STK* accumulate at the expense of their respective functional transcripts. The data presented here provide compelling evidence for considering the HUA-PEP proteins as part of the co-transcriptional regulatory machinery involved in coordinating transcription and pre-mRNA processing, and further highlights the importance of precise RNA regulation for correct plant reproductive morphogenesis.

## Introduction

Ovules are fundamental for plant reproductive success and food production. Ovule development predetermines the female gametophyte, proper fertilization, and ultimately fruit growth, as well as embryo and seed development [1]. Importantly, seeds constitute the primary basis for human sustenance and in recent years they have had an increasing role in biofuel production [2].

In flowering plants, ovules arise as lateral organs from meristematic placental tissue that differentiates inside the carpels, the female flower organs that constitute the pistil or gynoecium [3,4]. It is therefore that organ specification is a key aspect of ovule development. Ovule identity largely depends on the concerted action of MADS-box transcription factors collectively defined as the floral D-activity [5,6]. In the reference plant *Arabidopsis thaliana* (Arabidopsis hereafter) the D-class comprises three genes, *SHATTERPROOF1* (*SHP1*), *SHP2* and *SEEDSTICK* (*STK*), that redundantly confer ovule identity [7,8], in addition to playing crucial roles during fruit patterning and dehiscence (*SHP1*, *SHP2*) and fertilization or in seed coat development (*STK*) [9–11]. Thus, in *shp1 shp2 stk* plants, ovules lose identity and convert into flower organ-like structures whereas single or double mutants develop essentially normal ovules [7,8].

*SHP1*, *SHP2* and *STK* are the closest paralogs of the floral C-function gene *AGAMOUS* (*AG*) [12,13], a selector homeotic gene pivotal for flower patterning. As stated by the iconic ABCE model, the combinatorial activity of four classes of transcription factors specify the identity of flower organs in a stereotypical pattern of concentric whorls of sepals (A+E), petals (A+B+E), stamens (B+C+E) and carpels (C+E), respectively [14–16]. For example, in Arabidopsis *AG* specifies carpel identity in the fourth whorl in concert with E-class *SEPALLATA* (*SEP1* to *SEP4*) genes [17,18]. Genetic and molecular evidence indicated that SEP and AG proteins are also required for ovule identity in addition to the D-factors (Reviewed in [19]). According to the floral quartet hypothesis, MADS domain proteins assemble into diverse organ-specific (including ovules) tetrameric complexes [20–22]. Therefore, disrupting the MADS-box monomer balance may alter the stoichiometry of the corresponding tetramer and, thus, change specificity. For example, in *shp1 shp2 stk* triple mutant plants, ovules develop as flower organs that show carpellar features [7,8]. This transformation was interpreted as a reconfiguration of the MADS-box complexes from E (SEP) and D proteins (specifying ovule fate), to those including only AG and SEP proteins, thus conferring carpel identity as in the fourth whorl [7,8,21].

Temporal and spatial regulation of floral homeotic gene expression has been studied in great detail at the transcriptional level (reviewed in [16,23]). However, and although its importance is becoming increasingly more relevant, post-transcriptional control of floral homeotic genes has not been studied in detail [24–26]. In eukaryotes, superimposed layers of post-transcriptional regulation are major determinants of gene expression [27,28]. Producing functional RNA involves a complex interplay between transcription and RNA processing activities in which numerous RNA-binding proteins (RBP) participate, assembling into multifunctional ribonucleoprotein (RNP) complexes that coat nascent transcripts [27,29]. In this regard, the carboxyl-terminal domain (CTD) of RNA polymerase II (RNAP II) plays a pivotal role coordinating transcription and transcript maturation, thus increasing the fidelity of the process [27,29,30]. Modulation of the CTD, mainly via phosphorylation, is key to pre-mRNA co-transcriptional modifications, thereby affecting the final output of gene expression [29,31,32].

Processing of pre-mRNA via splicing and 3’ cleavage/polyadenylation expands the transcriptome and the proteome by generating multiple isoforms that increase developmental flexibility and adaptive responses of organisms [33–35]. This is particularly relevant for sessile organisms such as plants. Studies on plant differential RNA processing have been focused mainly on floral timing (recently reviewed in [28]) and plant-environment interactions [36,37]. However, the understanding of how pre-mRNA maturation impacts plant morphogenesis is still at its infancy. In Arabidopsis, the *HUA-PEP* activity [26] comprises a suite of RBP-encoding genes that genetically and physically interact to maintain the floral C-function by securing the correct processing of the *AG* pre-mRNA [24,26]. The *HUA-PEP* activity includes *HUA1*, which encodes a nuclear CCCH-type zinc-finger [38], the RPR-domain (Regulation of nuclear pre-mRNA) gene *HUA2* [39], and three KH (K-homology) domain genes: *HUA ENHANCER 4* (*HEN4*) [24], *FLOWERING LOCUS K* (*FLK*) [40,41], and *PEPPER* (*PEP*) [42]. Single loss-of-function mutants in *HUA-PEP* activity genes are essentially indistinguishable from wild-type plants. Conversely, higher-order *hua-pep* mutant combinations exhibit defects in floral organ identity and meristem determinacy that closely resemble those of *ag* mutants [12,24,26]. In line with this, *hua-pep* mutants accumulate aberrant and non-functional *AG* transcripts that are prematurely polyadenylated in the large second intron at the expense of the functional *AG* mRNA [26].

Here, we report that, in addition, the *HUA-PEP* activity controls ovule development by regulating the expression of D-class floral homeotic identity genes. Strong *hua-pep* mutants exhibit ovules transformed into flower organ-like structures and reduced levels of *SHP1*, *SHP2* and *STK* functional messengers, concomitant with the accumulation of aberrant transcripts prematurely terminated at intronic sequences. We also provide compelling evidence that the *HUA-PEP* activity can regulate their target genes even when they are mis-expressed outside the flower, supporting the fidelity and specificity of this regulation. Our data support a model in which HUA-PEP factors regulate RNA processing co-transcriptionally, a view reinforced by the ability of PEP and HUA1 proteins to interact with the CTD regulator C-TERMINAL DOMAIN PHOSPHATASE-LIKE1 (CPL1)/FIERY2 (FRY2) [43]. This study expands the functional scope of the *HUA-PEP* activity, and provides new insights into ovule development, illustrating the importance of co-transcriptional processing as a major gene regulatory mechanism in reproductive plant morphogenesis.

## Results

### The *HUA-PEP* gene activity affects ovule identity in Arabidopsis

**Fig 1:**
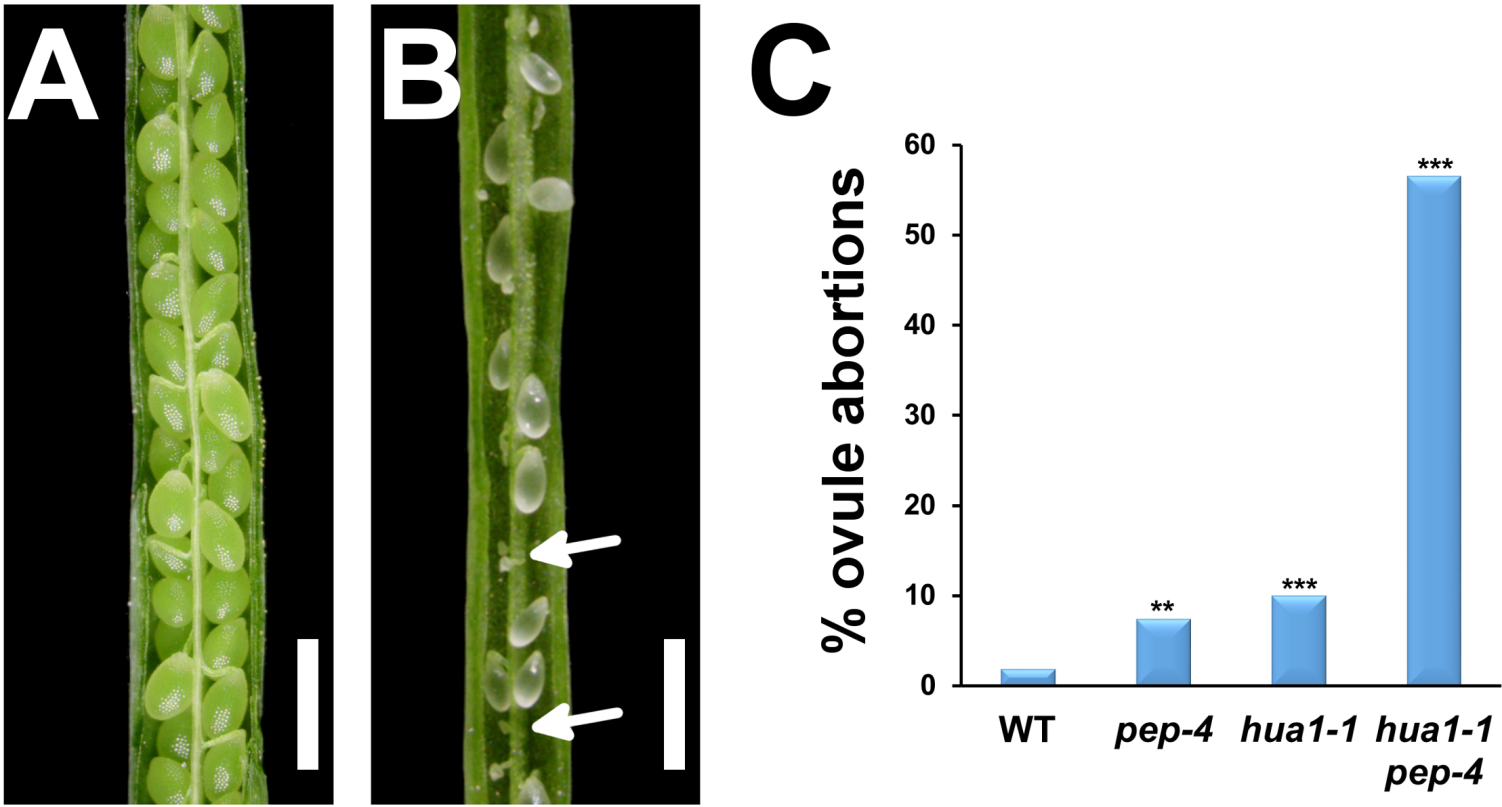
Ovule abortions reduce fertility in the *hua1-1 pep-4* mutant. A,B) Wild-type (A) and *hua1 pep* fruits (B) in which valve tissue was manually removed to show the rows of ovules/seeds in each locule. Ovule abortions in (B) appear as white tiny fists (arrows) evenly distributed along the length of the fruit ovary. C) Graphic representation of data shown in S1 Table. Scale bars: 1 mm. Asterisks indicate statistically significant differences from WT plants (** *P* < 0.01, *** *P* < 0.001).

Previous studies have shown that mutations affecting the *HUA-PEP* activity lead to dramatic morphological alterations in flowers [24,26]. In addition, sterility (or reduced fertility) was a recurring phenotype in *hua-pep* combinations, including genetic backgrounds in which flowers show minor or no obvious defects but yet producing fewer seeds than the wild type. For example, the *hua1-1 pep-4* double mutants are very weak when compared to stronger higher order *hua-pep* mutant combinations [26]. Nevertheless, they showed a significant loss of fertility due to reduction in seed set (S1 Table and Fig 1C), suggesting additional roles for the *HUA-PEP* gene activity besides flower morphogenesis.

**Fig 2:**
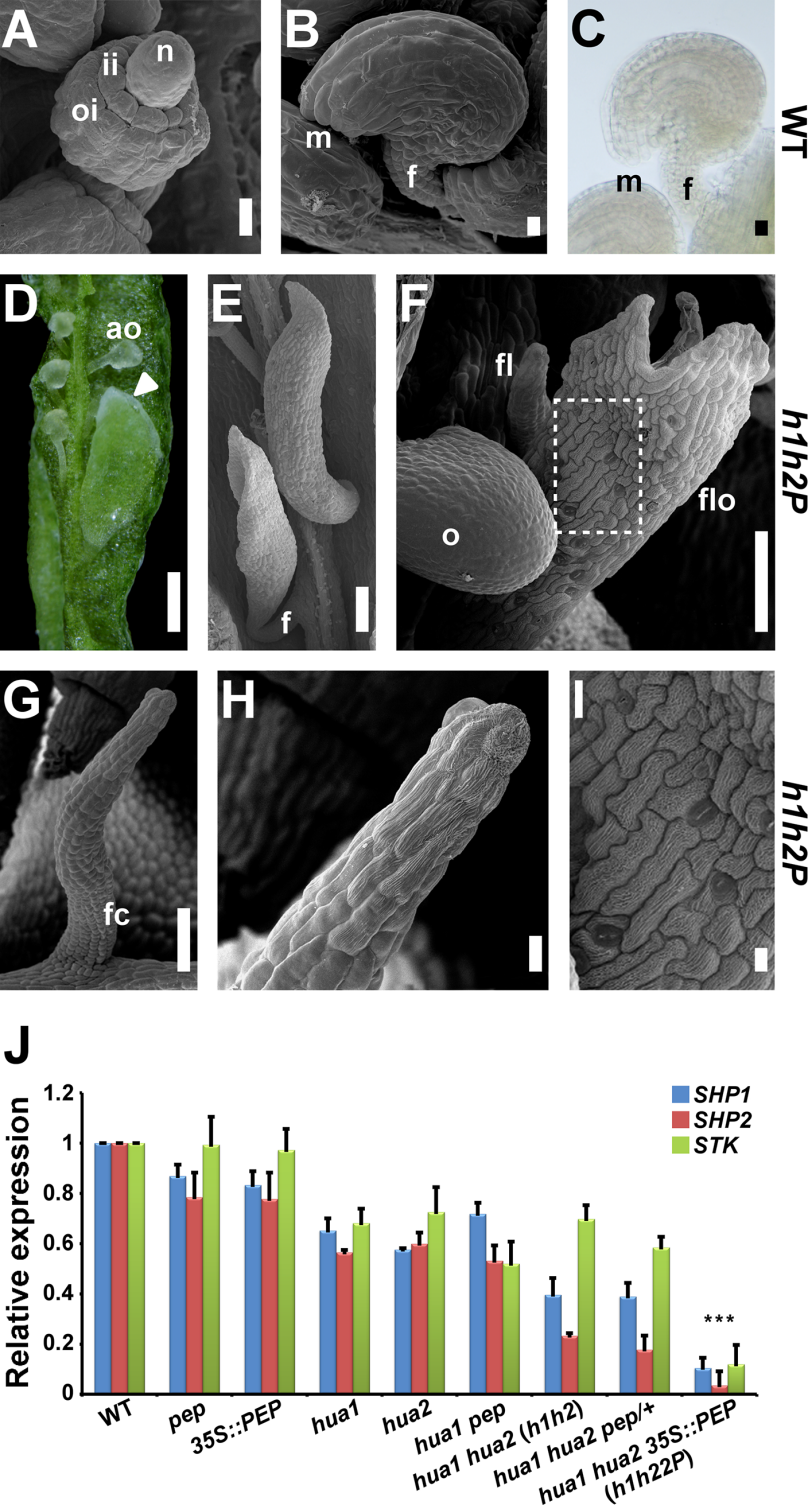
The *HUA-PEP* gene activity regulates ovule identity. A,B) Scanning electron micrographs (SEM) of developing (A) and mature (B) wild-type ovules. C) Light microscopy picture of a mature wild-type ovule. n, nucellus; oi, outer integument; ii, inner integument; m, micropyle; f, funiculus. D) Manually open *hua1 hua2 35S*::*PEP* (*h1h2P*) gynoecium showing aborted ovules (ao) and an ovule transformed into a leafy organ topped by white tissue (triangle). E-I) SEM images of *h1h2P* transformed ovules. E) Leafy organs with long funiculi (f). F) A normal-looking ovule (o) appears together with other ectopic structures resembling floral organs (flo) or finger-like (fl) protrusions. G) An ovule converted into a finger-like structure displaying funicular cells (fc) at the base. H) Close-up view of the organ shown in (G) in which crenulated cells can be observed on the apical portion. I) Close-up view of the area demarcated in (F) showing irregular wax-crenulated cells and interspersed stomata. Scale bars: 10 μm (A, B, C, H, I), 250 μm (D), 100 μm (E, F) and 50 μm (G). J) Relative transcript abundance of *SHP1*, *SHP2* and *STK*, monitored by quantitative RT-PCR (qPCR), in the wild type (WT) and diverse *hua-pep* mutant backgrounds. For simplicity, allele numbers (*pep-4*, *hua1-1*, *hua2-7*) have been omitted. Error bars denote standard deviation (SD). Asterisks indicate statistically significant differences from WT plants (*** *P* < 0.001).

We examined *hua1-1 pep-4* fruit and detected many empty spaces in the ovary corresponding to ovule abortions (Fig 1). Most interestingly, we observed that some ovules adopted floral organ identity (Fig 2 and S1 Fig). These ovule homeotic transformations occurred at a moderate frequency (~20% of flowers examined), being absent in single and most *hua-pep* double mutants, or with very low penetrance in *flk-2 hua2-4 pep-4* plants (5% of flowers; S2 Fig). However, in stronger mutant combinations such as *hua1-1 hua2-7* (~40%), *flk-2 hua1-1 hua2-7* (~80%), *hua1-1 hua2-7 pep-4/+* (93%) and *hua1-1 hua2-7 35S*::*PEP* (100%) their abundance was more conspicuous (Fig 2 and S1 Fig). The ectopic organs occurring in place of ovules showed very similar characteristics in the different *hua-pep* mutant combinations examined (see below).

As noted above, in the *hua1 hua2* background, either reduction (*hua1 hua2 pep/+*) or gain (*hua1 hua2 35S*::*PEP*) in *PEP* dosage lead to high profusion of ovule homeotic transformations and the same array of flower phenotypes [26]. Therefore, and unless indicated otherwise, *hua1-1 hua2-7* and *hua1-1 hua2-7 35S*::*PEP* plants (for simplicity, *h1h2* and *h1h2P* hereafter, respectively) were used as the reference genotypes to evaluate the effects of the *HUA-PEP* gene activity upon ovule identity.

Wild-type ovule primordia emerge from the placenta as finger-like outgrowths that later develop outer and inner integuments from the flanks of the chalaza to cover the distal nucella which contains the gametophyte (Fig 2A–2C). At maturity, full integument development leaves only a small opening, the micropyle, through which pollen sperm cells are discharged during fertilization. The ovule is connected to the placenta by a short stalk or funiculus (Fig 2B and 2C) [1,44]. By contrast, in *hua pep* backgrounds, transformed ovules often showed long funiculi and appeared as leaf-like organs with white or pale-green pointed tips (Fig 2D and 2E), reminiscent of the white fringe of tissue in sepals (S1E Fig). Close inspection by scanning electron microscopy (SEM) allowed us to verify that, rather than the typical smooth surface of wild-type ovule cells, in strong *HUA-PEP* activity mutant backgrounds the ovule surface contained wax-crenulated cells, irregular in size and shape, with interspersed stomata, which never form on ovules (Fig 2B, 2F and 2I). These morphological features are typical of sepal and carpel tissues, strongly suggesting that ovule integuments adopted sepaloid/carpeloid identity. In addition, ovules were sometimes replaced by finger-like protrusions that showed proximal funicular histology and distal cells with cuticular ridges (Fig 2F–2H). Altogether, these results evidence the importance of the *HUA-PEP* gene activity in ovule morphogenesis.

### The *HUA-PEP* gene activity targets the D-class genes (*SHP1*, *SHP2* and *STK*) for regulation

Members of the *HUA-PEP* gene activity regulate flowering time and flower morphogenesis by influencing the expression of the master regulatory MADS-box genes *FLC* and *AG* [24,26,40,41,45–48]. On the other hand, ovule identity is largely dependent on the MADS-box D-class genes *SHP1*, *SHP2* and *STK* and their closest paralog *AG* [7,8,13]. In this context, we decided to examine the effect of *hua-pep* mutations on the expression levels of the D-class genes using real-time quantitative PCR (qPCR). As shown in Fig 2J, transcript abundance of the three genes diminished, this reduction being more conspicuous as the severity of the *hua-pep* mutant phenotype increased. In line with this, *h1h2P* plants displayed the most dramatic ovule defects together with very reduced D-class gene transcript abundance (Fig 2J). This may explain the formation of long funiculi in these plants as a result of the reduced levels in *STK* expression, known to restrict funicular growth [8]. Pinyopich et al. [8] also described that in *shp1 shp2 stk* plants a fraction of ovules transformed into finger-like structures with radial symmetry; a defect that was also detected in *hua-pep* mutants (Fig 2F–2H).

In addition to their role during ovule morphogenesis, *SHP1* and *SHP2* are best known for their redundant role in valve margin differentiation and dehiscence (fruit opening) so that *shp1 shp2* fruit fail to dehisce and seeds get trapped inside the silique [9]. Interestingly, valve margin development in *h1h2P* fruit is blocked (S2 Fig), which explains the *h1h2P* indehiscent phenotype.

### Expression of D-class and *HUA-PEP* activity genes overlap in ovules

We previously reported that *PEP* is expressed in developing ovules [42]. This is not surprising as genetic backgrounds with compromised *PEP* expression, in combination with mutations in other members of the *HUA-PEP* activity lead to dramatic ovule defects (Fig 2 and S1 Fig). On the other hand, publicly available transcriptomic data show that the *HUA*-*PEP* activity genes (*PEP*, *FLK*, *HEN4*, *HUA1* and *HUA2*) are expressed in ovules [49].

**Fig 3:**
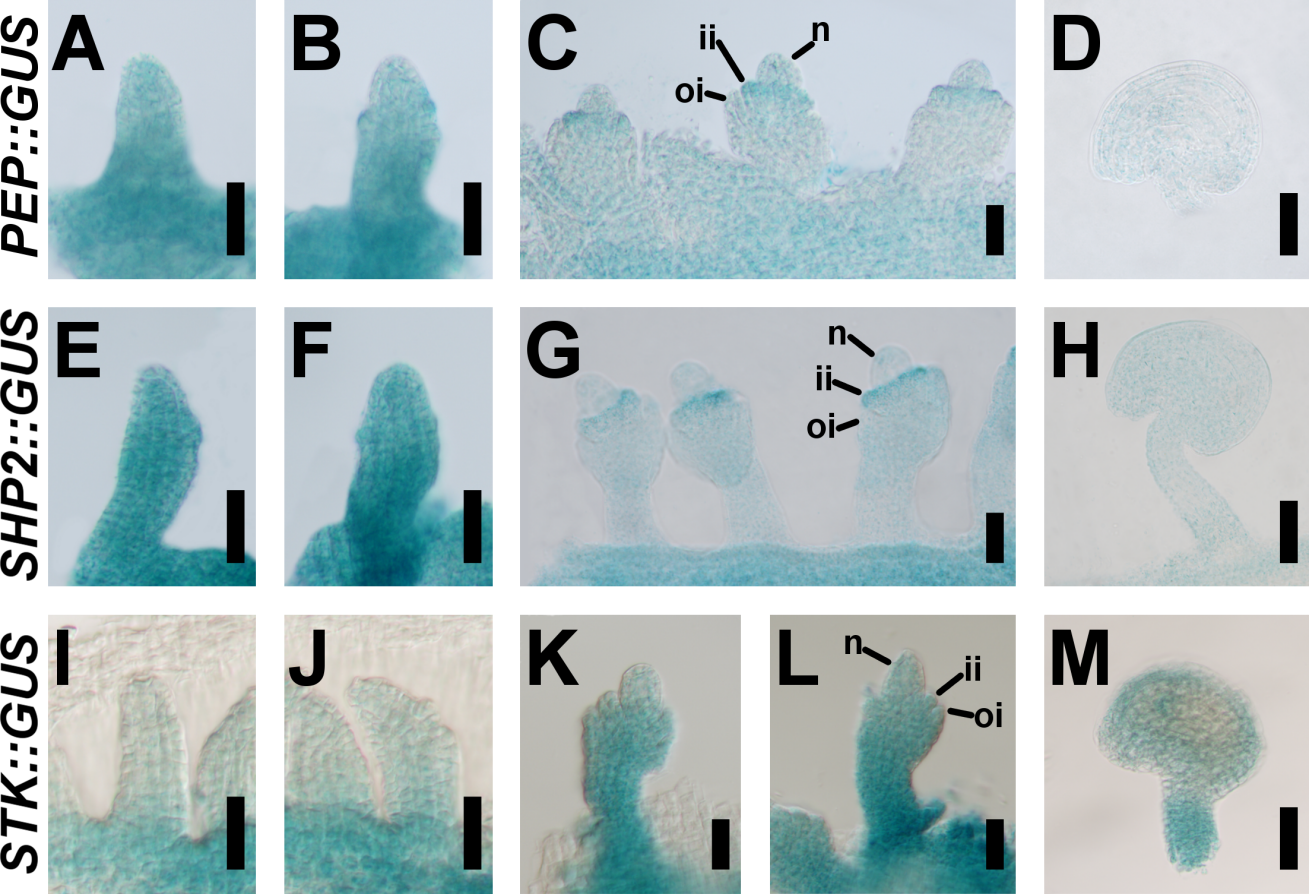
*PEP* and D-class gene expression patterns largely overlap during ovule development. Transcriptional reporter activity driven by the *PEP*::*GUS* (A-D), *SHP2*::*GUS* (E-H), and *STK*::*GUS* (I-M) constructs, during wild-type ovule development. *PEP* (A-D) and *SHP2* (E-H) reporter expression patterns are basically coincident. *STK*::*GUS* expression is more persistent in the funiculus and other territories of the ovule at later stages (K-M). n, nucellus; ii, inner integument; oi, outer integument. Scale bars: 25 μm (A-C, E-G, I-L) and 50 μm (D, H, M).

To gain further insight, we decided to use the *GUS*-reporter line *PEP*::*GUS* [42] as an expression “proxy” for the *HUA-PEP* activity, and compared its stage-by-stage ovule pattern to that of *SHP2* and *STK* marker lines. It is worth mentioning that the activity of these reporter lines essentially mirrors their respective mRNA *in situ* hybridization patterns [8,9,42,50]. *SHP1* was not analyzed as its expression pattern is virtually identical to that of *SHP2* [51,52]. The *GUS* signals for *PEP* and *SHP2* reporters were largely coincident during ovule development. At stage 2-I/II (all stages according to [44]), both reporters were broadly expressed in placental tissue and developing ovule primordia (Fig 3A, 3B, 3E and 3F). Later, at stage 2-III/IV *GUS* activity was higher in the growing inner integument (Fig 3C and 3G). At maturity, the signal became weaker in both cases (Fig 3D and 3H). In *STK*::*GUS* plants, reporter expression in funiculi was intense at stage 2-III/IV (Fig 3K and 3L), being the *GUS* activity more persistent than in the case of *PEP* and *SHP2* (Fig 3M). These results recapitulate previous reports for *SHP2* and *STK* expression during ovule development [8,52]. Our genetic and molecular data together support a model in which *HUA-PEP* function is active in ovules and targets the D-function genes for correct ovule morphogenesis.

### A genome-wide profiling approach using severe *hua-pep* mutant combinations uncovers altered expression of genes involved in flower and ovule morphogenesis

To generate a comprehensive view of the gene expression landscape influenced by the *HUA-PEP* activity, we performed RNA sequencing (RNA-Seq) experiments using the Illumina HiSeq2500 platform (see Materials and Methods). RNA was isolated from wild-type, *h1h2* and *h1h2P* flower buds. Our RNA-Seq analysis pipeline (false discovery rate or FDR threshold of 5%) uncovered 72 and 210 genes expressed at significantly higher levels in *h1h2* and *h1h2P*, respectively, relative to the wild-type (Col-0, S1 Dataset). Of these genes, 35 were common to *h1h2* and *h1h2P*. At this FDR level, 676 and 993 additional genes (including 502 common genes) were expressed at lower levels in *h1h2* and *h1h2P*, respectively, than in Col-0. The higher number of differentially expressed genes in *h1h2P* mutants when compared to *h1h2* strongly suggests that in the former the *HUA-PEP* activity is further compromised, which may explain the more dramatic phenotype of those plants. As expected from their genotypes, both *h1h2* and *h1h2P* had significantly reduced levels of At3g12680 (*HUA1*) and At5g23150 (*HUA2*), and *h1h2P* had significantly increased levels of At4g26000 (*PEP*) (S1 Dataset).

To generate a better view of the processes affected in *h1h2* and *h1h2P* plants, we searched for overrepresented gene ontology (GO) terms within differentially expressed genes, and performed Singular Enrichment Analysis (SEA) as implemented in the agriGO website (see Materials and Methods). We detected 24 and 48 GO terms significantly overrepresented in the sets of genes differentially expressed in *h1h2* (S3 Fig and S2 Dataset) and *h1h2P* (S4 Fig and S2 Dataset), respectively. Interestingly, some enriched GO terms were shared between *h1h2* and *h1h2P* sets, including terms such as ‘lipid localization’ (GO:0010876), ‘gametophyte development’ (GO:0048229), the related term ‘pollen development’ (GO:0009555), ‘floral whorl development’ (GO:0048438) and other terms specifically related to reproductive organ development. Some differentially expressed genes known to be required for gametophyte and/or floral whorl development included *ARGONAUTE 9* (*AGO9*; At5g21150), *AGAMOUS-LIKE 18* (*AGL18*; At3g57390), *ASYMMETRIC LEAVES 2* (*AS2*; At1g65620), *JAGGED* (*JAG*; At1g68480), *CRABS CLAW* (*CRC*; At1g69180), and *AG* (At4g18960), all downregulated in both *h1h2* and *h1h2P* (S1 Dataset). Other genes, including *NOZZLE*/*SPOROCYTELESS* (*NZZ*/*SPL*; At4g27330), *WUSCHEL* (*WUS*; At2g17950), *AUXIN RESPONSE FACTOR 17* (*ARF17*; At1g77850), *INNER NO OUTER* (*INO*; At1g23420), *SHP2* (At2g42830), and *STK* (At4g09960), were significantly downregulated only in *h1h2P* although their expression was also clearly reduced in *h1h2* (S1 Dataset).

**Fig 4:**
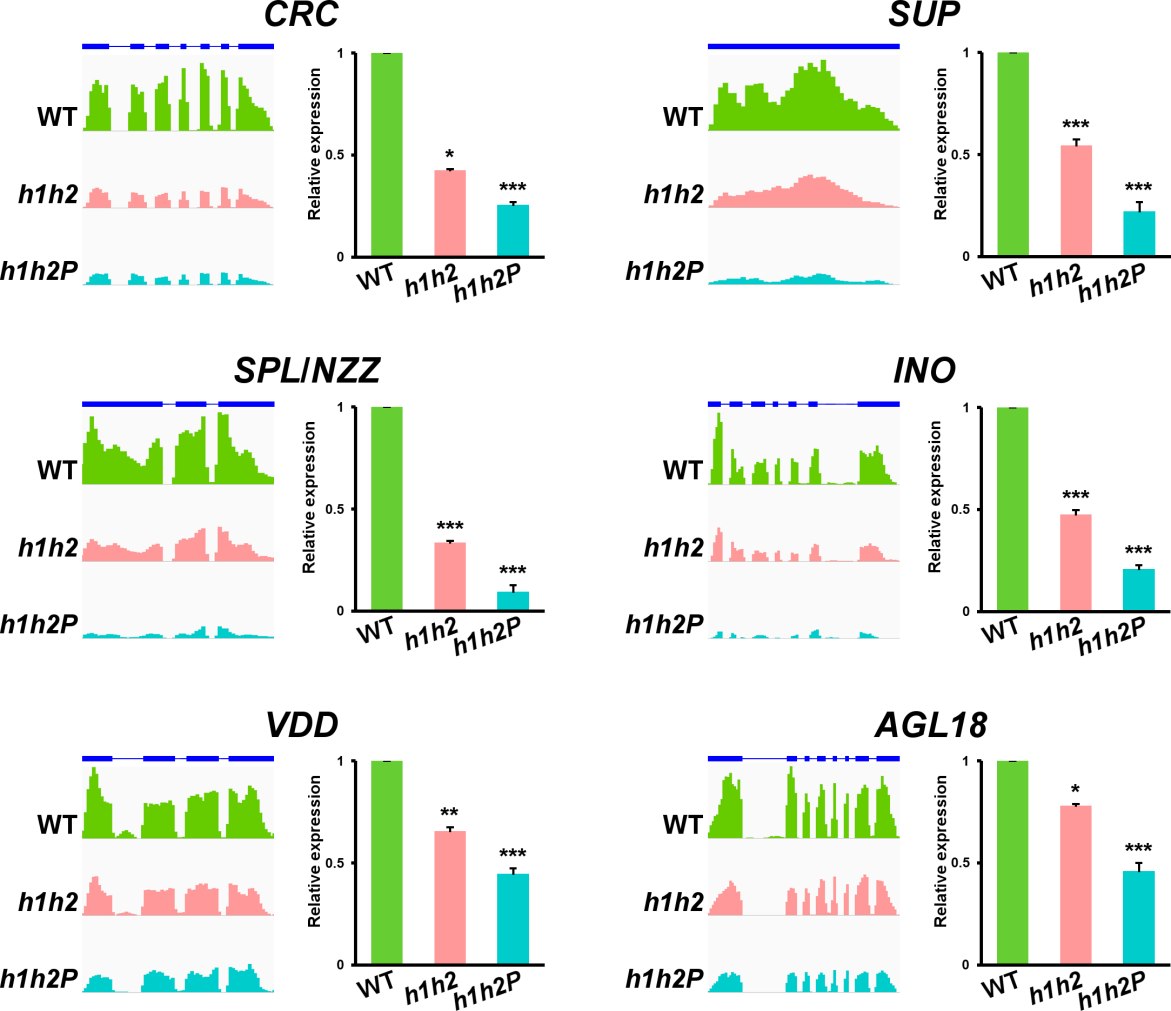
Validation of the RNA-Seq datasets. Quantification of gene expression levels of selected representative genes in Col-0 (WT) and *h1h2* and *h1h2P* mutant backgrounds. For each gene, RNA-Seq data (normalized read counts, as determined by IGV software [108]) are shown on left panels. Annotated gene structures are depicted on the top. Thick and thin bars represent exons and introns, respectively. On right panels, monitoring of gene expression levels by qPCR is presented. Error bars denote SD. Asterisks indicate statistically significant differences with respect to WT plants (* *P* < 0.05, ** *P* < 0.01, *** *P* < 0.001).

To confirm the accuracy of the transcriptomic profiling, we validated the expression of some of these genes using qPCR. In these studies, we also included genes participating in flower and/or ovule development whose variation was barely above the FDR threshold such as *SUPERMAN* (*SUP*, At3g23130) or *VERDANDI* (*VDD*; At5g18000). As shown in Fig 4 and S5 Fig, qPCR results largely mirrored RNA abundance inferred from RNA-Seq experiments. When compared to the wild type, RNA levels for these genes decreased in *h1h2* plants, being even lower in *h1h2P*, in agreement with the higher strength of this mutant background (Fig 4 and S5 Fig). Similarly, genes upregulated in the mutant backgrounds were more highly expressed in *h1h2P* than in *h1h2* plants (S5 Fig, S1 Dataset).

*CRC* provides carpel identity and contributes to style and stigma formation, and carpel fusion [23,53,54]. In addition to its role specifying boundary between whorls 3 and 4, *SUP* has been recently shown to be involved in keeping female identity and flower determinacy [55]. Thus, the reduced activity of these genes is consistent with developmental defects previously observed in *hua-pep* flowers [24,26]. Our results also indicated misregulation of genes critical for ovule development. *NZZ/SPL*, a direct downstream target of *AG* [51,56,57], was strongly downregulated in *hua-pep* mutants (Fig 4), providing further support and validation for our previous investigations [26]. Similarly, transcript abundance for *INO*, *VDD* and *AGL18* was also reduced in *hua-pep* mutants (Fig 4). *NZZ/SPL* promotes *INO* activity, which participates in integument development [19]. *NZZ/SPL* is also required for gametophyte development and, like *VDD*, known to work downstream of D-class genes and necessary for proper antipodal and synergid cell development [21,49]. *AGL18* is also expressed in developing gametophytes [58]. Taken together, these results support the notion that altering the *HUA-PEP* gene activity perturbs *AG* and D-class gene functions, and thus their downstream gene expression programs.

### The *HUA-PEP* gene activity facilitates correct pre-mRNA processing of D-class genes

We have previously shown that HUA-PEP proteins maintain the floral C-function via accurate processing of the *AG* large second intron. Otherwise, non-functional prematurely terminated transcripts, including intronic sequences, accumulate [24,26]. The genomic configuration of the D-class genes is similar to *AG*, containing long introns located near the 5’ end of the gene [59]. To test whether *HUA-PEP* factors affect precise processing of such intronic sequences, we examined the normalized read coverage for *SHP1*, *SHP2* and *STK* genes (including introns). For robustness, we incorporated *AG* into the analysis as a positive control.

**Fig 5:**
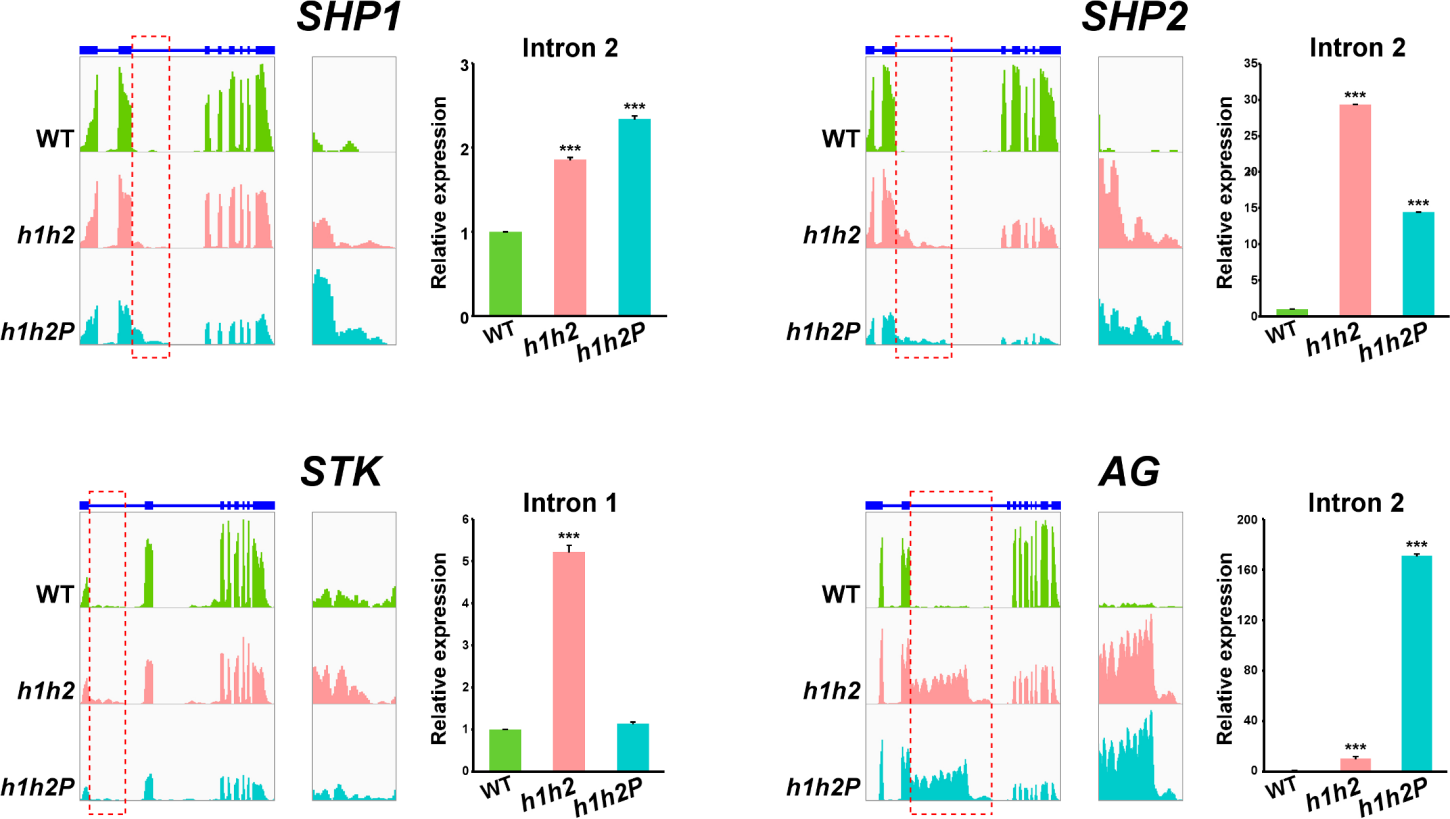
Pre-mRNA intron processing of D-class genes is impaired in strong *hua-pep* mutants. *SHP1*, *SHP2*, *STK* and *AG* transcripts abundance in Col-0 (WT), and *h1h2* and *h1h2P* mutant backgrounds. For each gene, RNA-Seq data (normalized read counts) are shown on left panels. Annotated gene structures are depicted on the top. Thick and thin bars represent exons and introns, respectively. Read coverage is represented according to the IGV software [108]. Intron read areas are demarcated by red frames, a magnification of which is shown on central panels. Right panels show relative expression levels, monitored by qPCR, of transcripts including sequences corresponding to introns 2 (*SHP1*, *SHP2* and *AG*) or 1 (*STK*). For *SHP1* and *AG*, to increase annealing specificity, forward primer sequences were split between exons 1 and 2 (see S2 Table for primers). Error bars denote SD. Asterisks indicate statistically significant differences with respect to WT plants (*** *P* < 0.001).

For *AG*, *SHP1*, *SHP2* and *STK*, the relative transcript abundance for exonic regions decreased in *h1h2* and *h1h2P* mutants when compared to wild-type (Fig 5), in line with the qPCR assays shown above (Fig 2J) and previous data on *AG* expression in the same mutant backgrounds [24,26]. It is worth noting that the RNA levels of *SHP1* (At3g58780) varied barely above the FDR threshold in our RNA-Seq experiments (S1 Dataset), even though our qPCR experiments firmly validated such changes (Fig 2J and see below).

Interestingly, we also observed reads mapping to the long second introns of *SHP1* and *SHP2* loci, as well as to the long introns 1 and 2 of *STK*. Intronic reads also appeared, although very scarcely, in the wild type and increased abruptly in the mutants (Fig 5). Such reads identified RNA products that extend beyond the exon-intron borders and terminate within the large introns, generating truncated and aberrant transcripts that exclude downstream exons and, thus, are not functional (Fig 5 and S6 Fig). Some of these transcripts were identified by 3’ rapid amplification of cDNA ends (3’ RACE) for the *SHP2* gene (S7 Fig). Indeed, in *h1h2* and *h1h2P*, transcript abundance for exons located after the long introns was lower than that of initial exons, indicating that the truncated transcripts account for most of the gene expression decrease observed in the mutants (Fig 5 and S6 Fig). This situation was most evident in *AG*, further confirming our previous observations (Fig 5; [26]).

To validate these findings we performed qPCR assays using RNA from wild-type, *h1h2* and *h1h2P* flower buds, and intronic primers located near the exon2/intron2 junction within the *SHP1* and *SHP2* loci, and exon1/intron1 junction within *STK* (see S2 Table for a list of primers). *AG* was again included as a positive control [26]. In all four cases, relative abundance of qPCR products incorporating intron sequences (corresponding to aberrant transcripts) increased significantly in the mutants when compared to wild-type, with the exception of *STK* intron 1 in the *h1h2P* mutant (Fig 5). This was also observed in our RNA-Seq results (Fig 5), in stark contrast with the dramatic reduction of the corresponding processed transcripts (see Fig 2J above). The levels of D-class gene transcripts were also estimated by measuring correctly spliced products corresponding to exons situated at the 3’ regions, downstream of their respective large introns (S8D Fig). Again, the three genes showed reduced expression in the mutant backgrounds (S8A–S8C Fig) conforming to upstream premature transcript termination. Altogether, these results support a role for *HUA-PEP* activity in intron processing, and further suggests that proper removal of long proximal introns seems to be a key regulatory aspect for *AG* and the D-class genes during development, critical for functional mRNA formation. It is worth noting that we detected a similar behavior (abnormally high number of reads in initial introns) in a few non-MADS-box genes of unknown function in the *hua-pep* mutant backgrounds (S9 Fig). However, the significance of this result is currently unclear.

### The *HUA-PEP* activity regulates floral genes in vegetative tissues of the *clf* mutant

Our current and previous studies have shown that the *HUA-PEP* activity targets *AG* and the D-class genes for correct transcript processing during reproductive growth ([26]; this work). In this scenario, we wanted to independently test the ability and robustness of the *HUA-PEP* activity to control these genes in developmental contexts in which *AG* and the D-class genes are not usually expressed. To this end, we made use of the *curly leaf* (*clf*) mutant. *CLF* encodes a component of the Polycomb repressive complex PRC2 that prevents the ectopic expression of floral homeotic genes (such as those forming part of the ABCDE model) outside the flower. Consequently, floral homeotic genes are ectopically expressed in *clf* rosette leaves, tissues in which normally floral homeotic genes are not expressed [60,61]. We therefore introduced the null *clf-29* Col-0 allele [62] into *h1h2*. We decided to use this background because, unlike *h1h2P* plants, *h1h2* plants are not fully sterile [26], thus facilitating the analysis.

**Fig 6:**
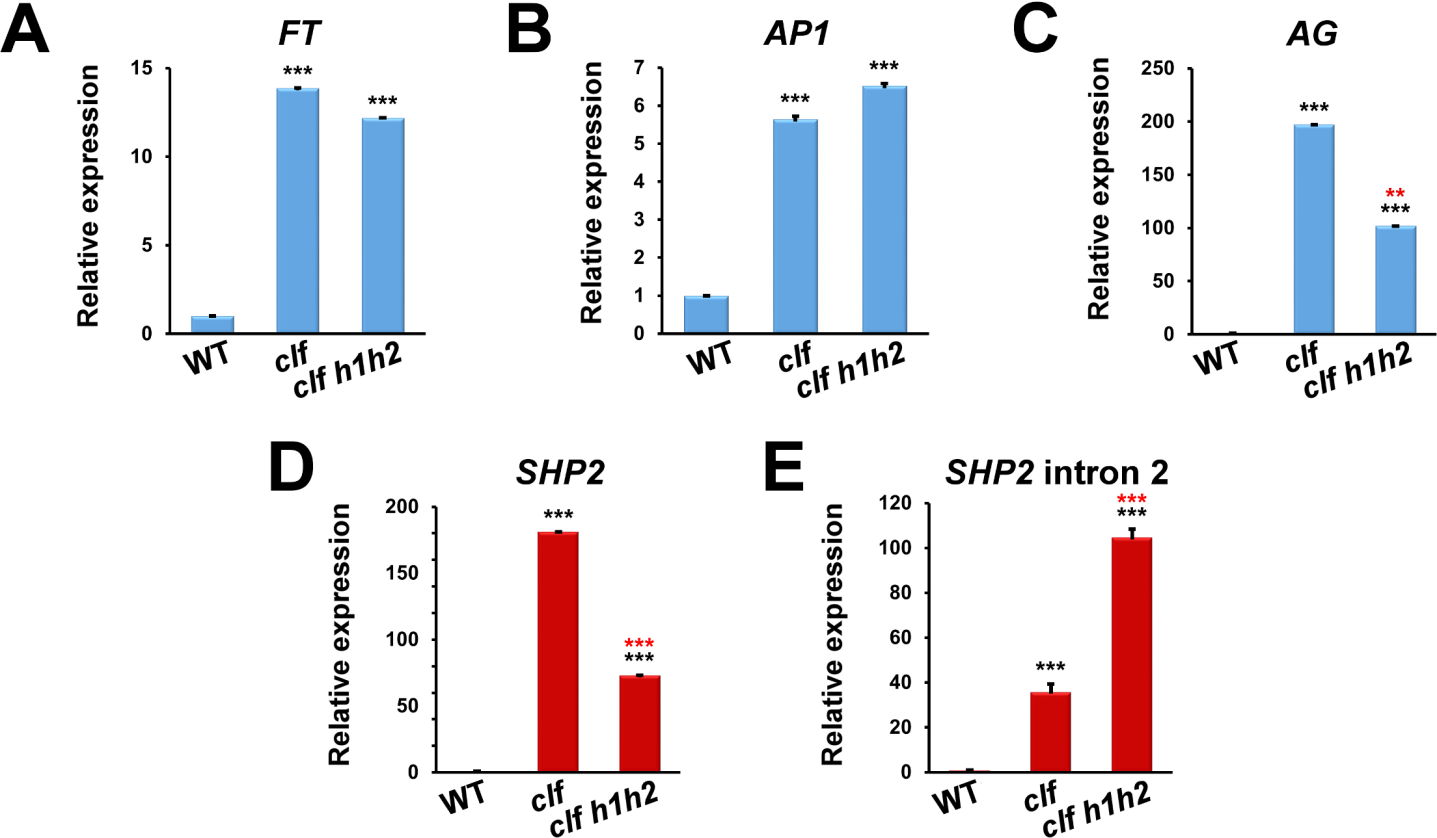
The *HUA-PEP* activity affects *AG* and *SHP2* ectopic expression in vegetative leaves. RNA was extracted from 10-day-old wild-type (WT), *clf-29* and *clf-29 h1h2* plants, and relative expression levels were monitored by qPCR. Relative expression levels of *FT* (A), *AP1* (B), *AG* (C) and *SHP2* (D) in *clf* rosette leaves. Functional *AG* (C) and *SHP2* (D) mRNA expression was determined by measuring levels of correctly spliced large intron 2. E) Relative expression levels of *SHP2* transcripts including sequences corresponding to intron 2. The same PCR primers as those corresponding to assays shown in Fig 2J and Fig 5 for *SHP2* were used for panels (D) and (E), respectively (S2 Table). Error bars denote SD. Black asterisks indicate statistically significant differences with respect to WT plants (*** *P* < 0.001). Red asterisks indicate statistically significant differences with respect to *clf-29* plants (*** *P* < 0.001, ** *P* < 0.01).

In addition to the ectopic expression of floral homeotic genes [60], CLF negatively regulates the transcription of the flowering integrator *FLOWERING LOCUS T* (*FT*), and thus, *clf* plants bolt precociously due in part to the high levels of *FT* expression [61,63] as we observed in *clf-29*. *FT* levels were not significantly different when comparing *clf-29* to *clf-29 h1h2* triple mutants (Fig 6A). Likewise, the up-regulation in *AP1* expression was very similar between *clf-29* and *clf-29 h1h2* plants (Fig 6B). This aligns with our previous observations showing that the *HUA-PEP* function has little or no effect on *AP1* control [26]. As expected and previously shown, *clf-29* plants also showed extremely high levels of *AG* ectopic expression [60] (Fig 6C). Interestingly, *AG* transcript abundance was much lower in *clf-29 h1h2* leaves (Fig 6C), reinforcing the idea that the *HUA-PEP* activity acts as a positive regulator of *AG* function ([24,26]; this work). Similarly, we observed that whereas *SHP2* transcripts were not detectable in wild-type leaves, they were highly abundant in *clf-29* samples and, interestingly, sharply attenuated in *clf-29 h1h2* rosette leaves (Fig 6D). We next monitored the relative abundance of *SHP2* transcripts retaining intron 2 sequences (see Fig 5 above). This category of transcripts was barely detectable in the wild-type leaves, whereas they accumulated in *clf-29* plants (Fig 6E). Remarkably, the relative amount of these aberrant transcripts further increased dramatically in the rosette leaves of *clf-29 h1h2* seedlings (Fig 6E). Altogether, these data reinforce our hypothesis that *HUA-PEP* activity targets *AG* and the D-class genes for regulation, and it does so regardless of the developmental context.

### The PEP and HUA1 proteins interact with the CTD phosphatase CPL1

**Fig 7:**
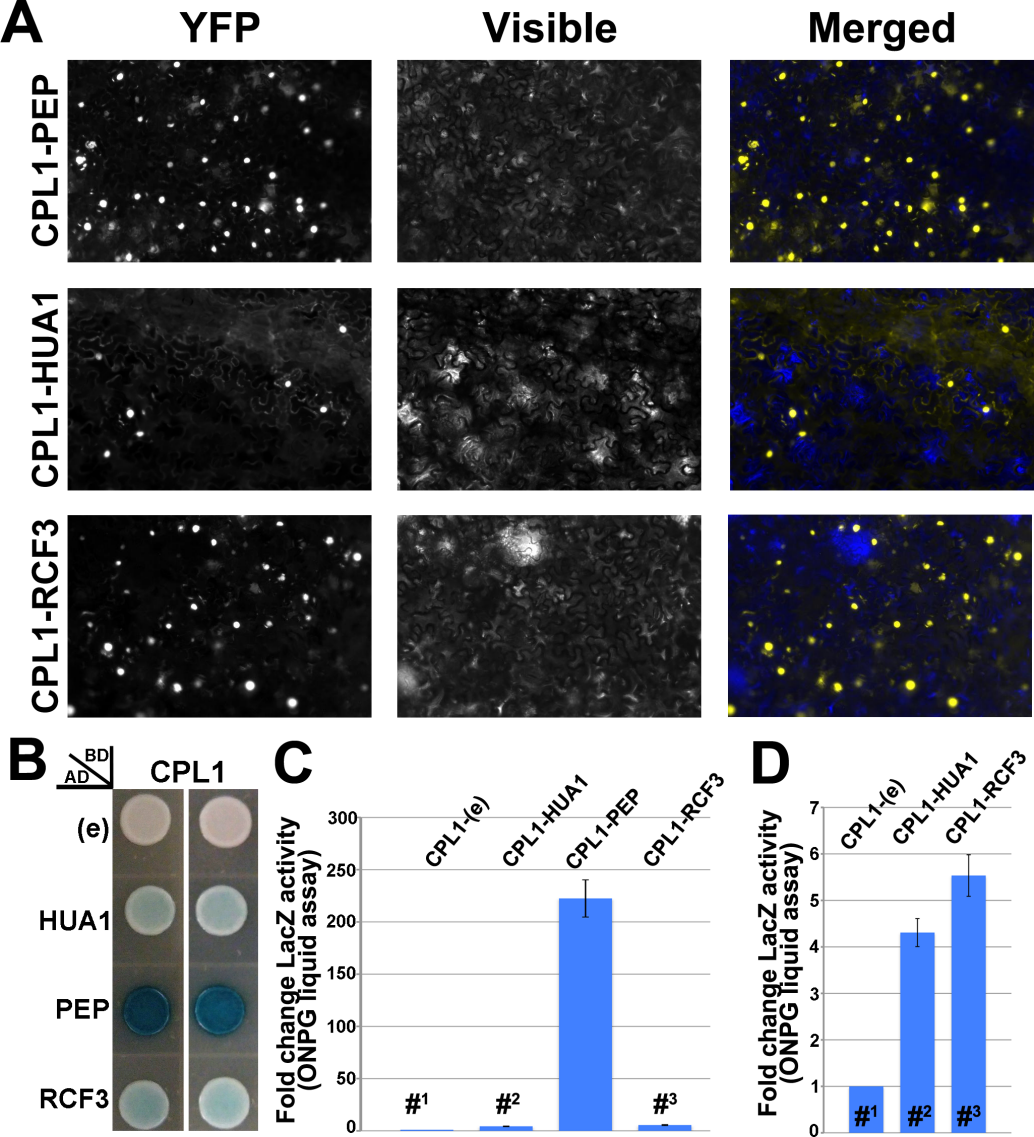
PEP and HUA1 physically interact with CPL1. A) BiFC visualization of protein dimerization (yellow fluorescence) in *Nicotiana benthamiana* leaf cells agroinfiltrated with plasmids encoding fusion proteins. In each test, the first protein was fused to the C-terminal fragment of the YFP (YFPct), and the second protein to the N-terminal portion (YFPnt), respectively (see Materials and Methods section). CPL1-RCF3 assays were used as positive controls. In merged visible+YFP fluorescence pictures, blue background was used to increase contrast. B-D) Y2H studies on plates (B) and liquid (β-galactosidase assays) (C,D), respectively. As in BiFC assays, RCF3 (known to associate to CPL1) was used as positive control. D) Magnification of the Y2H liquid assays performed in C for the CPL-empty vector (e; #^1^), CPL1-HUA1 (#^2^) and CPL1-RCF3 (#^3^) assays respectively.

Coordination of transcription and RNA processing is accomplished by the RNAP II CTD, whose phosphorylation status is critical in determining its activity [30,31]. In Arabidopsis, the CTD phosphatase FRY2/CPL1 plays a prominent role modulating co-transcriptional pre-mRNA processing thus affecting growth and stress responses [43]. Recently, a paralog of *PEP*, the KH-domain protein REGULATOR OF GENE EXPRESSION 3 (RCF3), aka HIGH OSMOTIC STRESS GENE EXPRESSION 5 (HOS5)/SHINY1 (SHI1)/ ENHANCED STRESS RESPONSE 1 (ESR1), has been identified as a CPL1 direct interactor [64–67]. On the other hand, the results shown above argue that the *HUA-PEP* activity affects D-class genes pre-mRNA processing co-transcriptionally. Therefore, we decided to test whether members of the HUA-PEP activity were capable of associating with CPL1. To this aim, we carried out *in vivo* and *in planta* protein-protein interaction assays. Both bimolecular fluorescence complementation (BiFC) assays and yeast-two-hybrid (Y2H) assays showed that PEP and CPL1 interact (Fig 7 and S10 Fig). We additionally challenged HUA1, a non-KH member of the HUA-PEP complex, against CPL1 and found interaction (Fig 7 and S10 Fig). Taken together, these results are consistent with the physical association of CPL1 with PEP and HUA1 proteins, strongly suggesting a functional interplay between *HUA-PEP* and RNAP II (likely via its CTD) activities, which is consistent with HUA-PEP proteins participating in pre-mRNA processing co-transcriptionally, probably influencing the phosphorylation status of the RNAP II CTD.

## Discussion

### The *HUA-PEP* gene activity regulates D-class gene expression protecting ovule identity

Development relies on precise mechanisms of gene regulation among which mRNA processing plays a critical role. We previously defined the *HUA-PEP* activity [26] as a post-transcriptional regulatory module composed by different RBPs that function in vital developmental programs for plant reproduction such as flowering time control and flower morphogenesis, by regulating the expression of *FLC* and *AG*, respectively [24,26,40,41,45,47,48]. This study uncovers an additional key contribution of the *HUA-PEP* activity in plant morphogenesis: the control of ovule development and identity by regulating the expression of the D-class homeotic genes [7,8,21]. We provide several lines of evidence based on molecular, genetic and genome-wide profiling analyses to support our model.

Mutant combinations affecting the *HUA-PEP* activity displayed homeotic transformations of ovules into floral organ-like structures similar to those described for D-class mutants [7,8]. Accordingly, loss of ovule identity was accompanied by a reduction in *SHP1*, *SHP2* and *STK* functional mRNAs. This was most obvious in the strongest *h1h2P* background, in which extremely reduced expression of the three genes nicely correlated with the high penetrance of the ovule homeotic conversions. The overlapping expression patterns between the D-class identity genes and the *HUA-PEP* activity genes is consistent with this regulation [42,49] (this study). Furthermore, by using the *clf* mutant background we have shown that the *HUA-PEP* activity retains its ability to regulate its target genes even when they are expressed ectopically in leaves.

The absence of valve margin in *h1h2P* gynoecia (S2 Fig) nicely fits with the down-regulation of *SHP1* and *SHP2* in our RNA expression assays (Fig 2J) [9]. The *SHP* genes also function in style formation and apical carpel fusion in concert with *CRC* [54]. Hence it is tempting to speculate that loss of *SHP* activity, together with the abatement of *CRC* expression (this study), contributes to the distorted open gynoecia previously described in *hua-pep* mutants [26].

**Fig 8:**
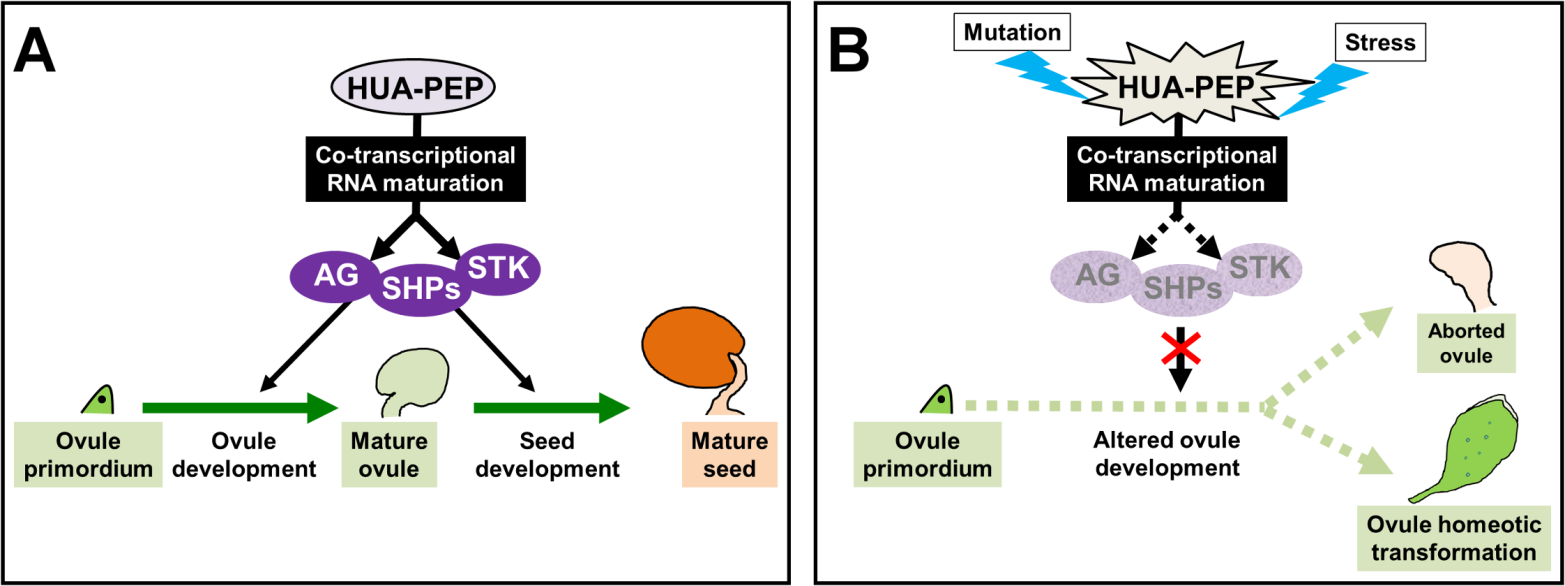
Hypothetical model for the regulation of ovule development by RNA processing. Proposed model for the influence of the *HUA-PEP* activity on ovule development upon environmental constraints. A) The HUA-PEP activity proteins facilitate pre-mRNA processing and hence production of functional AG and D-class proteins (purple circles). Under favorable conditions, AG, SHPs and STK are produced at a high rate, enough to properly regulate development of ovules (AG, SHPs, STK) and seeds (SHPs, STK). B) Upon mutation of the *HUA-PEP* activity genes, and possibly under stress conditions, premature transcript termination of *AG* and D-class gene pre-mRNAs might result in a critical decrease of transcripts encoding the corresponding functional proteins. Then, adequate development should be disrupted and instead ovule abortion occurs. Further reduction of ovule identity gene activities should account for homeotic transformations of ovules into flower organ-like structures.

Despite not being ovule-specific, our RNA-Seq study reveals that important genes critical for ovule patterning and function are misregulated in *hua-pep* mutant backgrounds. For instance, downregulation of *NZZ/SPL* and *VDD* is consistent with our model since they are both directly activated by *AG* and the D-class genes [49,56]. Moreover, *NZZ/SPL* promotes *INO* and *PHABULOSA* (*PHB*) expression in the ovule, and lack of *NZZ/SPL* perturbs the coordination between proximal-distal and adaxial-abaxial growth, also contributing to the appearance of longer funiculi (reviewed in [19]). In addition to homeotic transformations, low levels of *NZZ/SPL* may be related to ovule abortion in *hua-pep* mutants (Fig 8 and see below) since ovule formation is arrested in *nzz/spl* mutants early in development in Arabidopsis and tomato [68]. Likewise, *VDD* is required for proper female gametophyte development [49] like other genes that appeared down-regulated in our mutants, such as *AGL91*, *AGL87* and *AGL77* [69]. Finally, our qPCR and RNA-Seq assays allowed us to verify mRNA processing defects of D-class genes in *hua-pep* mutants, very similar to those previously described for *AG* ([24,26] and see below).

### Pre-mRNA processing of D-class genes is impaired in *hua-pep* mutants

Aside from their idiosyncratic protein modules, several MADS-box genes, including *AG*, *STK*, *FLC* or its close relative *FLOWERING LOCUS M* (*FLM*), contain large introns that house critical regulatory cis-elements conserved across species [70–77]. Lengthy introns, however, may increase the risk of aberrant mRNA processing due to cryptic signals. In yeasts, splicing of nascent transcripts was found to coincide with intron exit from RNAP II [78], and a recent study in Arabidopsis revealed the presence of numerous processing factors in the RNAP II elongation complex [79]. Given the structural similarity of *SHP1*, *SHP2*, and *STK* to *AG*, we conceived a role for the *HUA-PEP* activity in the maintenance of D-function by affecting processing of their corresponding pre-mRNAs, as previously shown for *AG* [26]. This idea gained support from our genome-wide data. Indeed, our RNA-Seq studies in strong *hua-pep* mutants corroborated the reduction of presumptive functional mRNAs for *AG* and D-class genes and the concomitant accumulation of aberrant transcripts (prematurely terminated within the large introns). These observations were validated by our PCR assays and, remarkably, were also substantiated by the analysis of ectopic expression in *clf* leaves. Moreover, we detected genes that, in the mutant backgrounds, also accumulated transcripts prematurely terminated inside large introns, which suggests that the regulatory action of the *HUA-PEP* activity might include functions other than securing the correct expression of the *AG*-clade members (S9 Fig). This is an issue worth to be explored in future studies.

### The *HUA-PEP* RNA processing activity might affect gene expression via interaction with the RNAPII CTD phosphatase CPL1

The results discussed above fit our previous model in which the HUA-PEP factors facilitate transcription elongation by preventing accessibility of the processing machinery to intronic cryptic signals in the nascent RNA, thus avoiding the production of non-functional transcripts [26]. In eukaryotes, coordinating transcription and RNA processing is an efficient mechanism to optimize gene expression during development. Co-transcriptional RNA modifications are surveilled by the CTD of the RNAP II large subunit, whose activity largely depends on its phosphorylation status [30,32]. We have shown that HUA1 and PEP proteins are binding partners of the phosphatase CPL1, a critical CTD regulator [43]. These results might support the participation of the *HUA-PEP* activity during co-transcriptional regulation of gene expression. A possible mechanism by which the HUA-PEP proteins protect nascent transcripts might involve their interaction with particular RNA sequence and/or structural motifs. Alternatively, but not mutually exclusive, the HUA-PEP proteins might modulate the activity of CPL1, and perhaps other phosphatases, critically affecting CTD phosphorylation and mRNA co-transcriptional modifications. Pre-mRNA splicing and 3’ end maturation occur co-transcriptionally, and the phosphorylated CTD bridges these processes by binding components of both processing machineries [29,30]. This is important because CTD phosphorylation increases protein accessibility to the elongation complex, and compactness may prevent imprecise spatiotemporal recruitment of processing factors [80]. Another possibility is that the HUA-PEP proteins might interact directly with the CTD to regulate proper incorporation of such processing factors.

In our protein-protein assays PEP showed a strong affinity for CPL1. PEP belongs to the group of KH proteins defined by mammal hnRNP K and PolyC binding protein (PCBP) family [42,81,82]. The KH domain can bind DNA and RNA, and can serve as a platform for protein-protein interactions [81]. RCF3, a previously identified CPL1 interactor, also contains KH domains similar to those of PEP [42,65]. Interestingly, the *rcf3* mutant displays altered polyadenylation site selection and intron retention [64,66]. Thus, it might be worth exploring the connection between *RCF3* and the *HUA-PEP* gene activity. However, those studies go beyond the scope of the current work.

### Developmental regulation by premature transcript termination

Alternative splicing and polyadenylation are widely accepted as basic mechanisms that add complexity, regulatory robustness and flexibility to animal and plant genomes [28,35]. Additionally, in animal systems, regulation of prematurely processed transcripts is emerging as an important checkpoint to modulate developmental and adaptive decisions in a tight cross-talk with splicing [83–87]. KH-domain proteins seem to play prominent roles in these two regulatory processes. Thus, knock-down of mammalian PCBPs, KH-domain proteins structurally related to PEP, favors usage of cryptic intronic processing sites and the accumulation of non-effective transcripts for pre-mRNAs and long non-coding RNAs (lncRNA) [81,88,89]. Remarkably, binding of the KH-domain splicing factor Sam68 to an intronic polyadenylation site of the *Aldehyde Dehydrogenase 1A3* (*Aldh1a3*) gene prevents its recognition and premature transcript termination, thus promoting self-renewal of mouse neural progenitor cells rather than differentiation [90].

In plants, flowering time regulation provides examples of developmental switches based on the use of intronic polyadenylation sites. Thus, the levels of FCA and FPA functional proteins, two *FLC* regulators, are controlled through a negative feedback by premature polyadenylation in their long third and first intron, respectively [91,92]. Intronic polyadenylation in the large first intron of the floral repressor *FLM* was also detected in the wild type, being suggested as a mechanism for modulating *FLM* transcript levels [93], thus contributing to adapt floral timing to optimal conditions. In fact, this type of regulation has been recently demonstrated to modulate ambient temperature-dependent flowering in natural Arabidopsis accessions [76,77]. Although at very low levels, prematurely terminated *AG* and D-class gene transcripts are also present in the wild type ([24,26]; this study) and, beyond the floral transition, reproductive development can be perturbed by adverse circumstances that reduce fertility. For instance, ovule abortion increases under stress [94,95]. The *hua-pep* mutants also show ovule abortions, indicating that compromising the *HUA-PEP* activity affects ovule viability without altering identity (Fig 8). Indeed, *shp1 shp2 stk* triple mutant plants are virtually sterile. Although not all of their ovules are homeotically transformed, they show instead numerous abortions [8]. *AG* and D-class genes are fundamental to ovule identity acquisition but they are also necessary to activate gene functions required for further development of maternal and gametophytic tissues [49,96–98]. Stress conditions might impinge upon the *HUA-PEP* activity, altering RNA processing of *AG* and D-class genes, thus affecting flower and ovule development (Fig 8). This could contribute to fine-tune the allocation of resources for reproduction and stress tolerance. Exploring this scenario surely deserves further investigation.

## Materials and methods

### Plant material

This work was carried out with the *Arabidopsis thaliana* Columbia (Col-0) accession as the wild type. Strains previously obtained in L*er*, *hen4-2* [24], *hua1-1* and *hua2-1* [39], were backcrossed at least five times into Col-0 before any further experiment. Other lines used in this study were *pep-4* and *PEP*::*GUS* [42], *flk-2* [41], *hua2-4* [45]; *hua2-7* [47], *35S*::*PEP* [46], *SHP2*::*GUS* [50], and *STK*::*GUS* [8]. *clf-29* (SALK_021003) was obtained from the NASC. Information about all primers used in this work and molecular genotyping can be found in S2 Table. Plants were grown in MS plates or soil as previously described [42].

### Microscopy and histology

Light microscopy and scanning electron microscopy (SEM) were performed as previously described [42]. Samples were also cleared with Hoyer solution [99] for 30 min and observed under differential interference contrast (DIC) optics. All *GUS* staining assays were performed in homozygous lines, essentially as described [42,100,101]. Light microscopy samples were photographed in a Nikon E800 microscope equipped with a Nikon Digital Camera DXM1200F (operated by the ACT-1 2.70 program).

### Quantitative PCR and RACE

For quantitative reverse transcriptase-polymerase chain reaction (qPCR), 5 μg of total RNA was extracted from young flower buds (until stage 9) or 10-day-old rosettes, treated with DNase I, and used for cDNA synthesis with an oligo(dT) primer and RevertAid Reverse Transcriptase (Thermo Fisher) following the manufacturer’s instructions. Subsequently, for each qPCR reaction, 0.5 μl of the cDNA was used as template. Relative changes in gene expression levels were determined using the LightCycler 1.5 system with the LightCycler FastStart DNA amplification kit according to the manufacturer (Roche Diagnostics). RNA levels were normalized to the constitutively expressed gene *OTC* (*ORNITHINE TRANSCARBAMYLASE*), and the corresponding wild-type levels, as previously reported [26]. Each experiment was undertaken using three biological replicates with three technical replicates each. Statistical significance was estimated by the Student’s *t*-test according to [102] (* *P* < 0.05, ** *P* < 0.01, *** *P* < 0.001).

3’ rapid amplification of cDNA ends (3’ RACE) was conducted as previously reported [26, 103]. 5 μg of young flower bud total RNA was reverse transcribed using Maxima Reverse Transcriptase and the adaptor oligo d(T)-anchor (kit 5’/3’ RACE, Roche Diagnostics) as a primer. Then, *SHP2* cDNAs were amplified with Phusion High-Fidelity DNA Polymerase (Thermo Scientific) using forward primers situated in the exon 2 (S2 Table) and the PCR anchor (Roche Diagnostics) as a reverse primer hybridizing with the adaptor sequence, thus ensuring that only polyA-containing sequences were amplified. Amplified products were cloned into pSC-A plasmids and sequenced with M13F and M13R primers. Sequences were analyzed using CLUSTAL-W aligning [104].

### RNA-Seq and bioinformatics analysis

Library construction was performed using the TruSeq Stranded mRNA Library Preparation Kit (Illumina) and the resulting fragments were sequenced in the lllumina Hiseq 2500 platform, using 100 bp paired-end reads, at StabVida (Caparica, Portugal). The bioinformatic analysis was performed as described in [105]. Paired-end reads were aligned to the TAIR10 version of the *Arabidopsis thaliana* genome sequence and annotation (https://www.arabidopsis.org/) using Tophat version 2.2.1 [106] and Bowtie 2 version 2.2.4.0 [107], feeding the program with the coordinates of TAIR10 gene models in a GFF (General Feature Format) file (using option -G) and discarding all discordant read mappings (with options—no-discordant and—no-mixed). Transcript levels were quantified for these gene models using the cuffdiff program of the Cufflinks version 2.2.1 package [106] after filtering out all reads mapping to rRNA, tRNA, snRNA and snoRNA genes, whose coordinates were supplied in a separate GFF file (using option -M). Two biological replicates were used for each genotype. The resulting read alignments, supplied as files in BAM format, were visualized using Integrative Genomics Viewer (IGV) [108] and Tablet software [109].

For the identification of overrepresented GO terms, we used the agriGO online tools (http://bioinfo.cau.edu.cn/agriGO/; [110]) using a selected set of genes (including those marked “OK” by Cufflinks) as the customized annotated reference, as previously described [111].

### Protein interactions

BiFC and Y2H were performed as previously described in [26] and [112]. For BiFC, the corresponding coding sequences were amplified from their respective cDNAs using the proof-reading Phusion (New England Biolabs, Inc.) polymerase (see S2 Table for primers) and cloned into pBJ36-SPYNE and/or pBJ36-SPYCE plasmids, containing N-terminal (nt) and C-terminal (ct) halves of the yellow fluorescent protein (YFP), respectively (YFPnt and YFPct) [113]. The resulting *35S*::*SPYNE* and *35S*::*SPYCE* cassettes were sequenced and then cloned into the T-DNA binary vectors pGreen0229 and pGreen0179 [114], respectively. Transformed AGL-0 *Agrobacterium tumefaciens* cells were used to infect *Nicotiana benthamiana* leaves. YFP reconstituted fluorescence was visualized 72 h after inoculation under a Nikon Eclipse TE2000-U epifluorescence microscope. The reciprocal BiFC assays were also performed obtaining the same results as shown in Fig 7 and S10 Fig, thus endorsing specificity of the interactions. As a positive control we used CPL1-RCF3 assays, previously shown to associate [64–66]. As negative controls, *Nicotiana* leaves were co-infiltrated with the corresponding recombinant YFPct construct and the empty YFPnt version. As additional negative interactions we assayed PEP, HUA1 and RCF3 against a non-related ARF transcription factor (see Fig 7 and S10 Fig, and reference [115].

For yeast two-hybrid assays, the cDNA PCR amplicons for *PEP*, *HUA1*, *CPL1* and *RCF3* genes were generated using the corresponding primers (S2 Table) and cloned into the pB42AD (+Trp) and pGilda (+His) vectors via Gibson DNA assembly procedure [116]. The integrity of the resulting pGilda and pB42AD constructs was checked by sequencing. The yeast strain EGY48 (-Ura) was cotransformed with the corresponding combinations of pGilda and pB42AD constructs. Empty vectors were used as negative controls. Positive colonies were selected on solid media (-Ura, -His, -Trp +glucose). Induction for testing protein-protein association was assayed growing the resulting yeast strains on plates in the presence of galactose and raffinose (DB Falcon). X-gal was used for colorimetric assays on plates (SIGMA), and ONPG (2-Nitrophenyl β-D-galactopyranoside, SIGMA) for β-galactosidase liquid experiments. The Clontech protocol book was followed for all these procedures.

## Supporting information

S1 Fig Additional examples of ovule transformations in *hua-pep* mutants. A-D) Manually open gynoecia of *hua1-1 pep-4* (A), *hua1-1 hua2-7 pep-4/+* (B), *flk-2 hua2-4 pep-4* (C), *flk-2 hua1-1 hua2-7* (D), *hua1-1 hua2-1 pep4/+* (F, G), *hua1-1 hua2-4 35S*::*PEP* (H) and *hua2-1 hen4-2 pep-4/+* (I). Ectopic leafy organs, aborted ovules (ao) and developing seeds (s) are shown. Since doubly null *hua2 pep* mutants are inviable, the leaky *hua2-4* allele [26] was used to construct the *flk hua2 pep* triple mutant shown in (C). Ovule homeotic transformations never occurred neither in *hua2-4 pep-4* nor in *hua2-7 pep-4/+* plants. E) Wild-type sepal showing a characteristic white fringe of tissue at the tip (arrow). Scale bars: 0.5 mm (A, C, D-G), 1 mm (B) and 250 μm (E).
(TIFF)

S2 Fig Valve margin development is disrupted in *h1h2P* mutant gynoecia. SEM (A-C) and cross sections (D,E) of Col-0 (A,D) and *h1h2P* mutant gynoecia (B,C,E) at later stages of development, showing the absence of valve margin in the mutant (red asterisks in E). r, replum; v, valve; vm, valve margin. Scale bars: 100 μm (A, C-E) and 10 μm (B).
(TIFF)

S3 Fig Directed acyclic graphs showing the relationships between overrepresented GO terms assigned to genes differentially expressed between Col-0 and *h1h2*. Three different graphs are shown, corresponding to terms belonging to the three main subontologies: (A) Cellular component, (B) Molecular function, and (C) Biological process. The false discovery rate (FDR) of significantly overrepresented GO terms is given in parentheses, and the corresponding graph nodes are filled in with different tones of yellow (less significant) to red (more significant). The frequency of each term in the set of differentially expressed genes and in the background set is also given, and matches the values on S2 Dataset.
(PDF)

S4 Fig Directed acyclic graphs showing the relationships between overrepresented GO terms assigned to genes differentially expressed between Col-0 and *h1h2P*. Three different graphs are shown, corresponding to terms belonging to the three main subontologies: (A) Cellular component, (B) Molecular function, and (C) Biological process. The false discovery rate (FDR) of significantly overrepresented GO terms is given in parentheses, and the corresponding graph nodes are filled in with different tones of yellow (less significant) to red (more significant). The frequency of each term in the set of differentially expressed genes and in the background set is also given, and matches the values on S2 Dataset.
(PDF)

S5 Fig Additional validation of the RNA-Seq datasets. Quantification of gene expression levels of selected representative genes in Col-0 (WT) and *h1h2* and *h1h2P* mutant backgrounds. For each gene, RNA-Seq data (normalized read counts, as determined by IGV software [108]) are shown on left panels. Annotated gene structures are depicted on the top. Thick and thin bars represent exons and introns, respectively. On right panels, monitoring of gene expression levels by qPCR is presented. Error bars denote SD. Asterisks indicate statistically significant differences with respect to WT plants (*** *P* < 0.001). *CAPRICE-LIKE MYB3* (*CPL3*) is involved in trichome branching and epidermal cell differentiation [117]. The *YABBY* family gene *FILAMENTOUS FLOWER* (*FIL*) is involved in abaxial tissue specification and participates in flower formation [118] and the mediolateral axis of the fruit [100, 101]. *JASMONATE-AMIDO SYNTHETASE 1* (*JAR1*) encodes the key conjugating enzyme that yields the bioactive form of jasmonate (JA), jasmonolyl-L-isoleucine (JA-Ile) [119]. *FLOWERING LOCUS M* (*FLM*) encodes a MADS-box polypeptide well known for its role as a flowering repressor, particularly in the thermosensory pathway [120,121]. *SQUAMOSA PROMOTER LIKE 8* (*SPL8*) and *SPL15* are SBP-box genes, members of the *SPL* family involved in various processes including flowering, stamen and sporogenesis development [122,123]. The basic helix-loop helix transcription factor-encoding *ABORTED MICROSPORES* (*AMS*) is essential for male fertility and activates the cytochrome P450 gene *CYP703A2* required for sporopollenin synthesis in the anther [124].
(TIFF)

S6 Fig *SHP2* transcripts prematurely terminated within intron 2 visualized with the Tablet software. Screenshots of read coverage tracks obtained from the Tablet software [109] in Col-0 WT (A) and *h1h2* (B) plants. On top of both panels, the annotated gene structure is depicted. Relative positions of exons (salmon-red bars) and intron 2 are indicated. Blue and green indicate forward and reverse reads that are properly paired according to the mapping software (Bowtie2 and Tophat). In the WT (A), abundant reads connecting exons 2 and 3 can be observed, indicative of correctly spliced exon 2. Read coverage corresponding to 3’-most exons is also abundant. In the *h1h2* mutant (B) less reads corresponding to proper intron 2 splicing are detected and numerous reads corresponding to interrupted transcripts appear. In line with this, reads covering the 3’-most part of the gene decrease dramatically with respect to the wild-type (red arrow). C) Schematic representation of terminated transcripts within the *SHP2* intron 2. Thick blue and red bars denote coding and non-coding exonic sequences, respectively. Thin bars represent introns. Prematurely terminated transcripts at different points inside intron 2 are represented by wavy lines partly orange in color. A solely red and blue wavy line symbolizes the fully mature mRNA encoding a functional polypeptide (thick blue bar below).
(TIFF)

S7 Fig Sequence scheme of prematurely processed *SHP2* transcripts identified by RACE. DNA sequence corresponding to exon 2 appears as white upper-case letters boxed in black. Intron 2 sequence is shown as lower-case black letters. Cleavage site is indicated (C in red). The sequence corresponding to the specific forward primer is underlined.
(TIFF)

S8 Fig D-class gene expression levels measured as correctly spliced transcripts produced at their 3’ regions. A-C) Relative expression levels, monitored by qPCR, of *SHP1* (A), *SHP2* (B), and *STK* (C) genes in wild-type plants (WT) and the *h1h2* and *h1h2P* mutant backgrounds. Expression levels were inferred from relative abundance of correctly spliced transcripts produced at their 3’ regions, located downstream from the respective large introns. D) Schematic diagram of an idealized gene representative of the three D-class members. Blue boxes denote exons whereas intronic regions are colored in orange. Relative positions of primers used for measurements in panels A-C (red arrows) are indicated (see S2 Table for specific primer sequences). Error bars denote SD. Asterisks indicate statistically significant differences with respect to WT plants (*** *P* < 0.001).
(TIFF)

S9 Fig Intron processing defects in non-MADS-box genes in strong *hua-pep* mutants. At1G33080, At3G05165 and At5G03610 transcripts abundance in Col-0 (WT), and *h1h2* and *h1h2P* mutant backgrounds. For each gene, RNA-Seq data (normalized read counts) are shown. Annotated gene structures are depicted at the bottom of each panel. Thick and thin bars represent exons and introns, respectively. Read coverage is represented according to the IGV software [108]. Intron read areas are separated from those corresponding to preceding exons by red vertical lines. At1G33080 encodes a protein predicted as a MATE efflux family protein, an integral component of membrane with transport activity. At3G05165 encodes a major facilitator family protein, a putative integral component of membranes involved in transport. AT5G03610 encodes a GDSL-motif esterase/acyltransferase/lipase. It belongs to an enzyme group with broad substrate specificity that may catalyze acyltransfer or hydrolase reactions with lipid and non-lipid substrates. Source, TAIR (http://www.arabidopsis.org/index.jsp).
(PDF)

S10 Fig Additional protein-protein interaction assays to test CPL1, RCF3, PEP and HUA1. Visualization of YFP reconstitution (yellow fluorescence) in *Nicotiana benthamiana* leaf cells agroinfiltrated with plasmids encoding fusion proteins. The first 3 interactions show the reciprocal assays of those depicted in Fig 7. The interaction between RCF3 and CPL1 [65,66] was used as a positive control. As negative controls, *Nicotiana* leaves were co-infiltrated with the corresponding recombinant YFPct construct and the empty YFPnt version. The reciprocal assays were also performed and, in both cases, no signal was detected [26]. An additional control was used in which HUA1 and PEP constructs were challenged against a B3 transcription factor from the ARF5 (Auxin Response Factor 5) in both orientations, and no YFP fluorescence reconstitution was observed in these experiments. In merged visible+YFP fluorescence pictures, blue background was used to increase contrast.
(TIF)

S1 Table Ovule abortions in *hua1-1*, *pep-4* and *hua1-1 pep-4* mutants. The table summarizes the data of two independent rounds of counting. “Total primordia” refers to the total number of ovules produced, regardless of their viability.
(PDF)

S2 Table Oligonucleotides, genotyping and additional references. (DOCX)

S1 Dataset Results from the RNA-Seq analyses in Col-0, *h1h2* and *h1h2P* samples. File in MS Excel format containing the results of the differential expression analysis in three separate tabs for (a) Col-0 versus *h1h2*, (b) Col-0 versus *h1h2P*, and (c) *h1h2* versus *h1h2P*. Each tab contains the normalized average expression values (expressed as FPKM; fragments per kilobase of gene per million fragments mapped) and the lower and upper limits of the confidence interval for each genotype, as determined by the Cufflinks software package.
(XLSX)

S2 Dataset Overrepresented GO terms identified by means of Singular Enrichment Analysis. File in MS Excel format containing the results of the SEA analysis (as implemented in the agriGO website) in two separate tabs for (a) overrepresented GO terms identified in the set of genes differentially expressed between Col-0 and *h1h2* samples, and (b) overrepresented GO terms identified in the set of genes differentially expressed between Col-0 and *h1h2P*. We assigned 970 different GO terms (116 in the “Cellular component”, 330 in the “Biological function”, and 524 in the “Molecular process” subontologies) to the 748 genes differentially expressed between Col-0 and *h1h2*, and 1397 terms (145 in the “Cellular Component”, 485 in the “Biological Function”, and 767 in the “Molecular Process” subontologies) to the 1203 genes differentially expressed between Col-0 and *h1h2P*. We used SEA to identify a subset of significantly overrepresented terms assigned to the differentially expressed genes. Twenty-four and 48 GO terms were significantly overrepresented in the sets of genes differentially expressed in *h1h2* and *h1h2P*, respectively, including terms from the Biological Process (16 and 33, respectively), Cellular Component (6 and 9) and Molecular Function (2 and 6) subontologies.
(XLSX)
