# Supplementary figures and images for: Ovule identity mediated by pre-mRNA processing in Arabidopsis
Source: PLoS Genet. 2018 Jan 12;14(1):e1007182. doi: 10.1371/journal.pgen.1007182 (PMC5785034; doi:10.1371/journal.pgen.1007182)

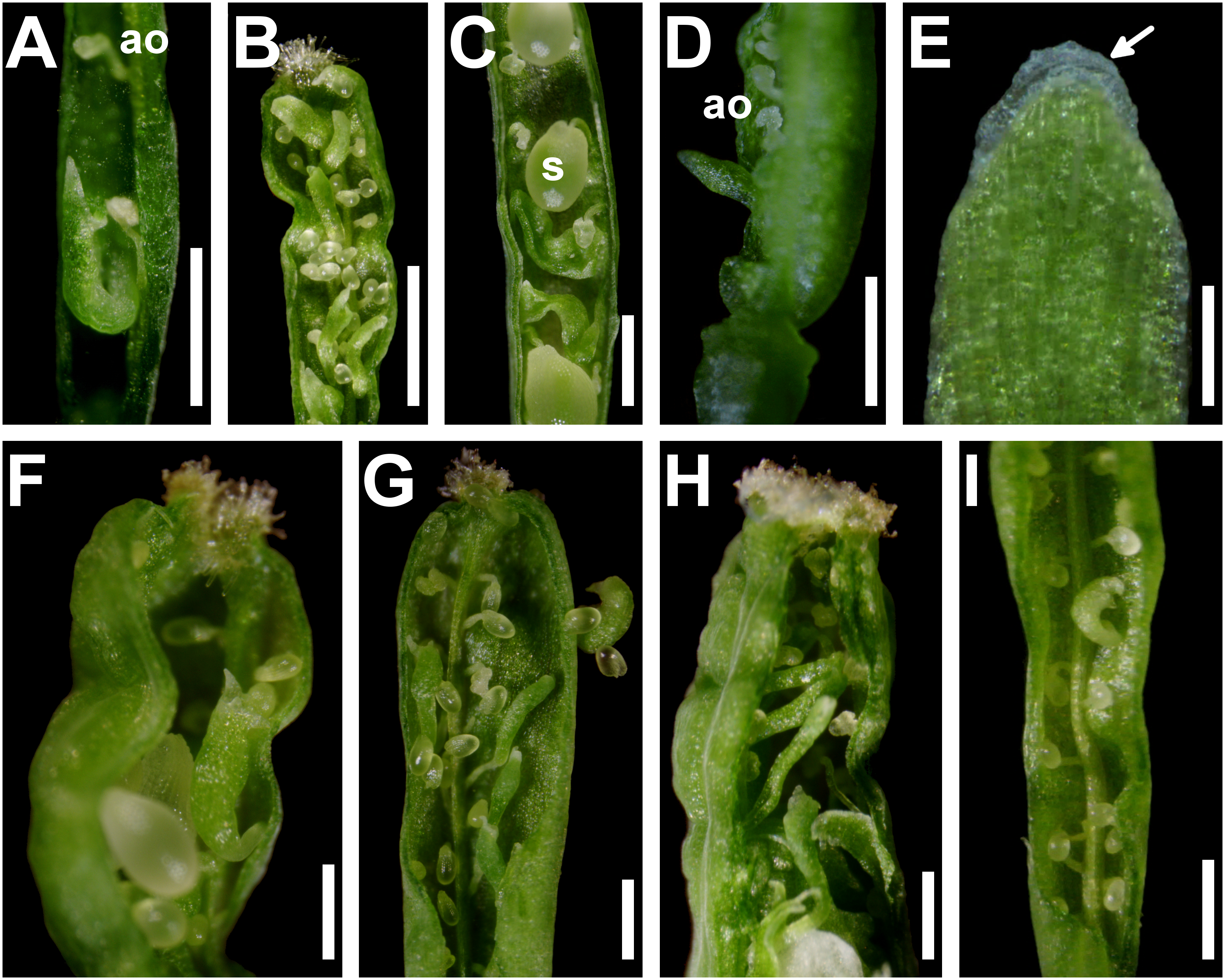

Supplement: S1 Fig — A-D) Manually open gynoecia of hua1-1 pep-4 (A), hua1-1 hua2-7 pep-4/+ (B), flk-2 hua2-4 pep-4 (C), flk-2 hua1-1 hua2-7 (D), hua1-1 hua2-1 pep4/+ (F, G), hua1-1 hua2-4 35S::PEP (H) and hua2-1 hen4-2 pep-4/+ (I). Ectopic leafy organs, aborted ovules (ao) and developing seeds (s) are shown. Since doubly null hua2 pep mutants are inviable, the leaky hua2-4 allele [26] was used to construct the flk hua2 pep triple mutant shown in (C). Ovule homeotic transformations never occurred neither in hua2-4 pep-4 nor in hua2-7 pep-4/+ plants. E) Wild-type sepal showing a characteristic white fringe of tissue at the tip (arrow). Scale bars: 0.5 mm (A, C, D-G), 1 mm (B) and 250 μm (E). (TIFF) [file pgen.1007182.s001.tiff]

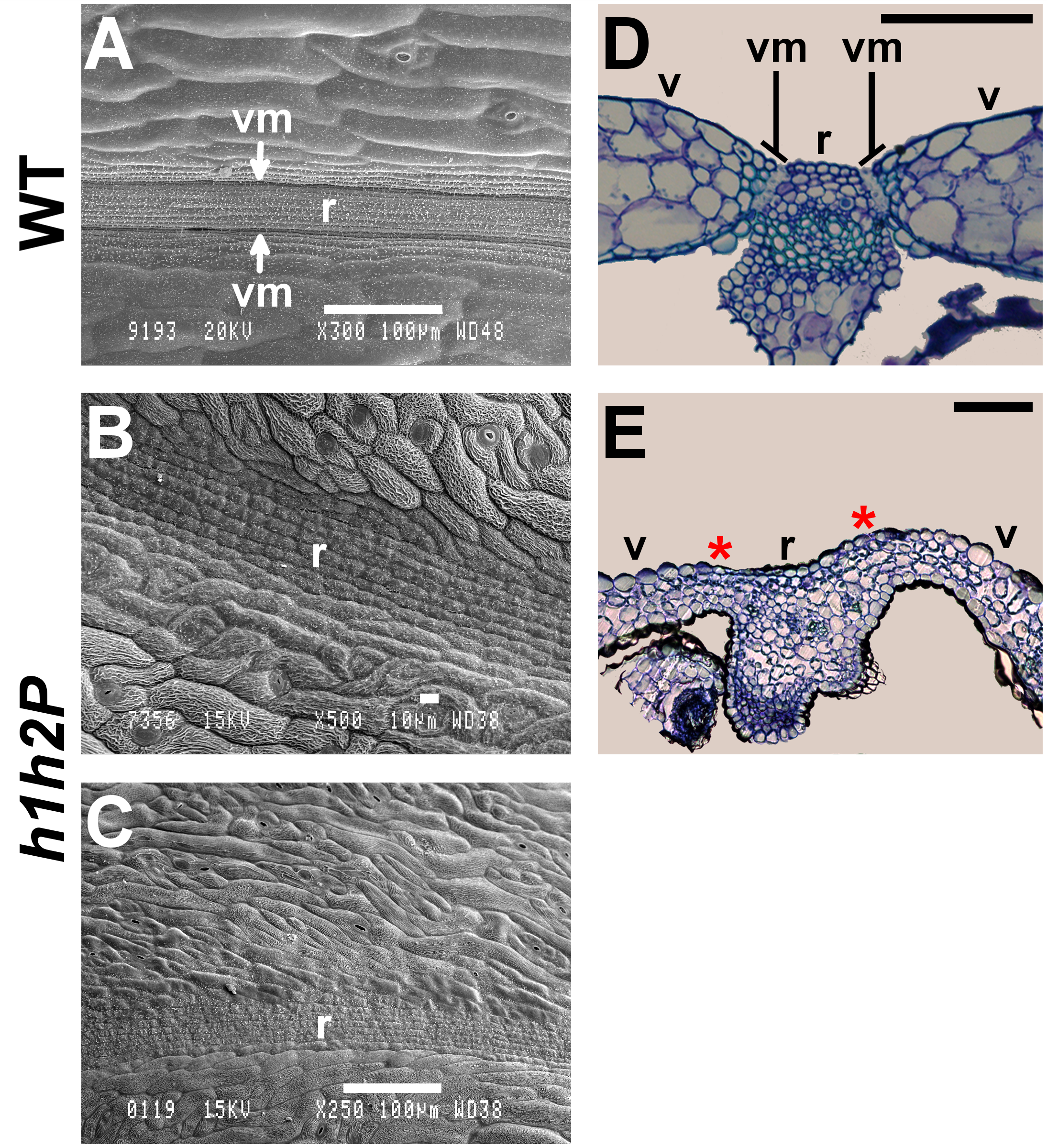

Supplement: S2 Fig — SEM (A-C) and cross sections (D,E) of Col-0 (A,D) and h1h2P mutant gynoecia (B,C,E) at later stages of development, showing the absence of valve margin in the mutant (red asterisks in E). r, replum; v, valve; vm, valve margin. Scale bars: 100 μm (A, C-E) and 10 μm (B). (TIFF) [file pgen.1007182.s002.tiff]

A

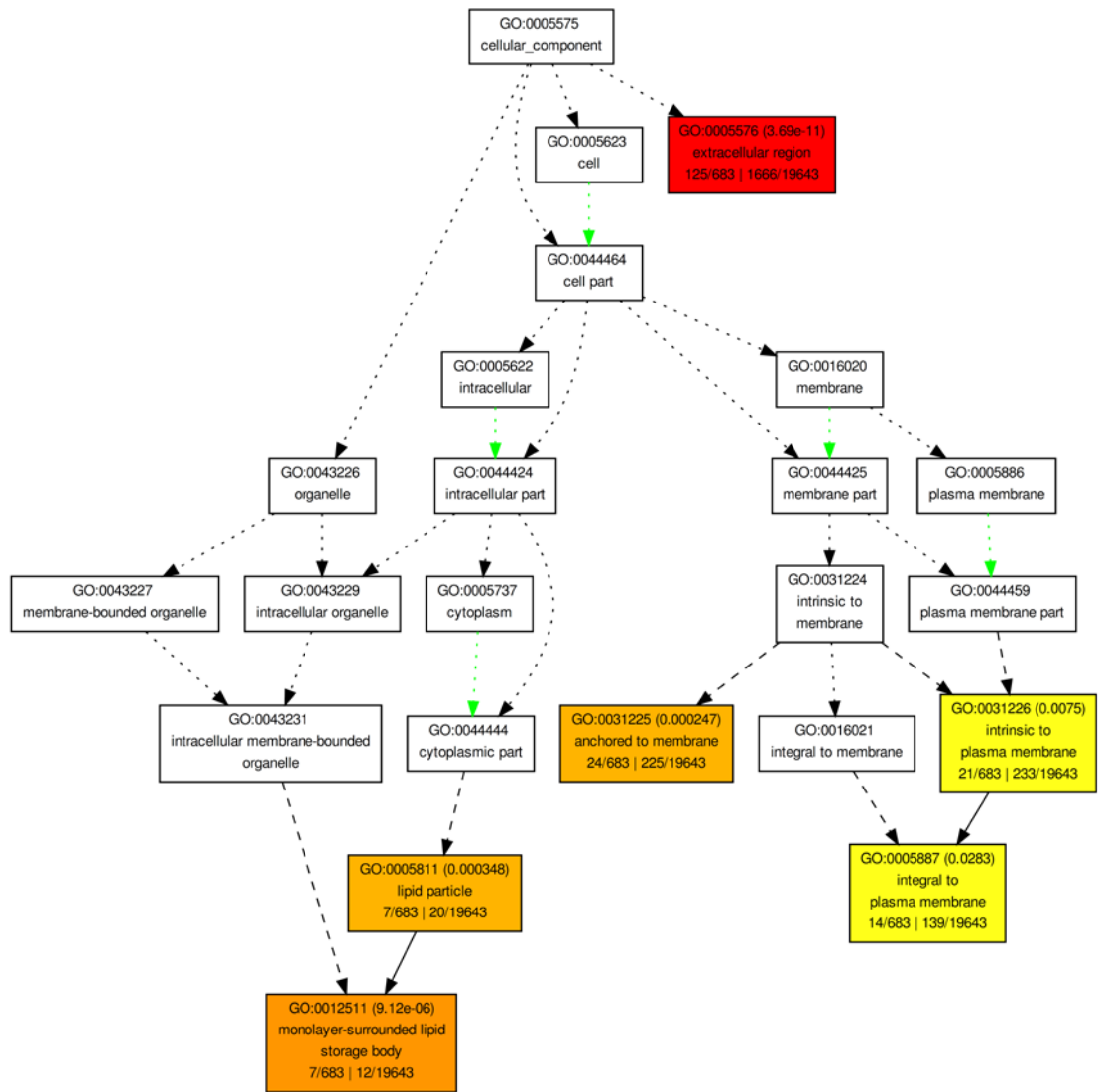

**B**

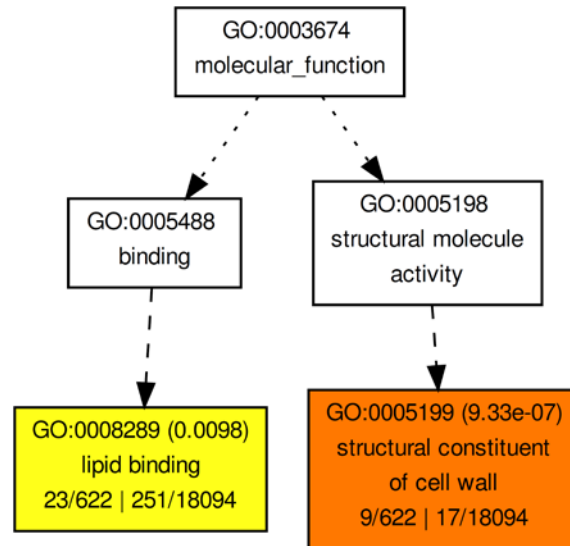

C

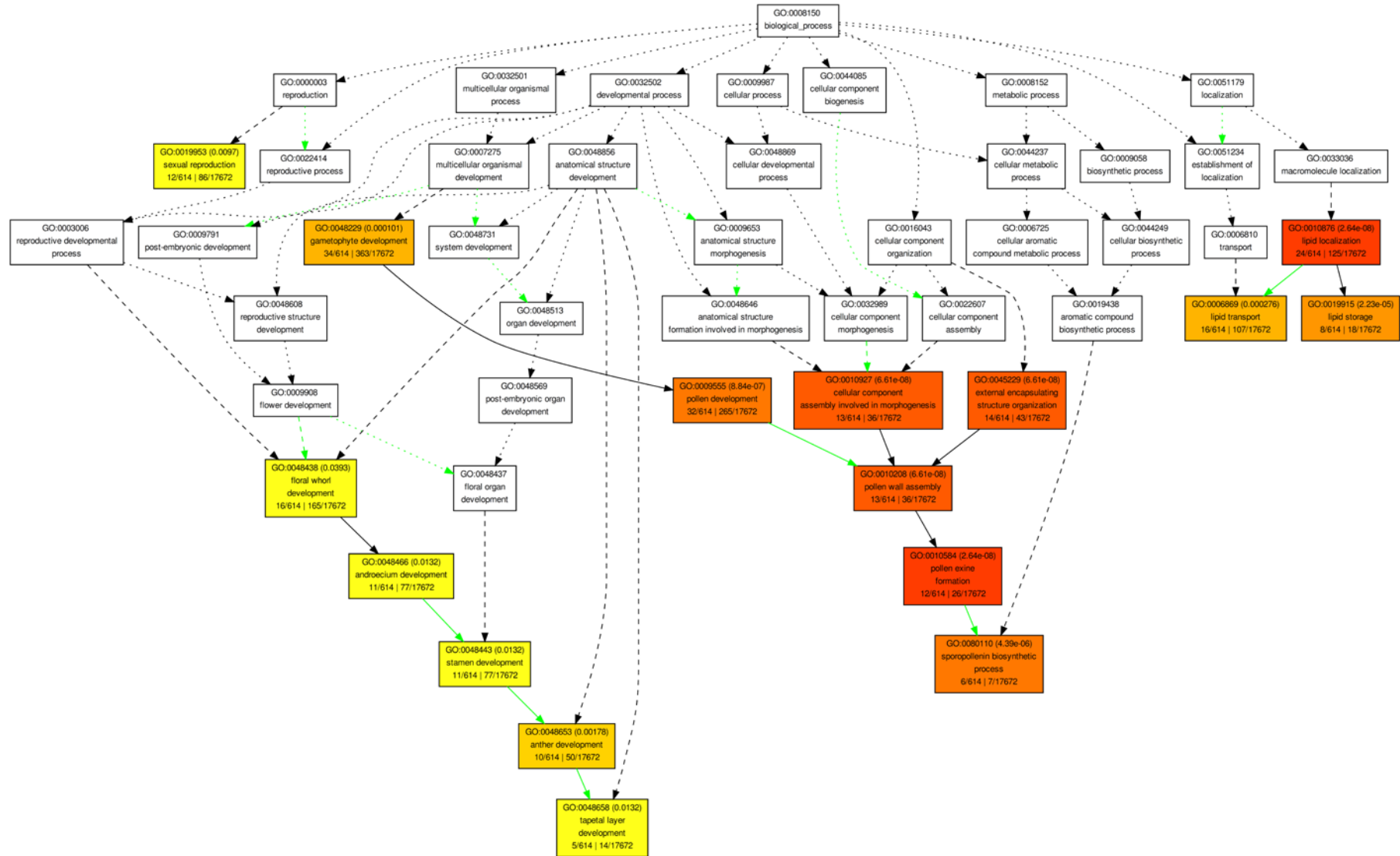

Supplement: S3 Fig — Three different graphs are shown, corresponding to terms belonging to the three main subontologies: (A) Cellular component, (B) Molecular function, and (C) Biological process. The false discovery rate (FDR) of significantly overrepresented GO terms is given in parentheses, and the corresponding graph nodes are filled in with different tones of yellow (less significant) to red (more significant). The frequency of each term in the set of differentially expressed genes and in the background set is also given, and matches the values on S2 Dataset. (PDF) [file pgen.1007182.s003.pdf]

A

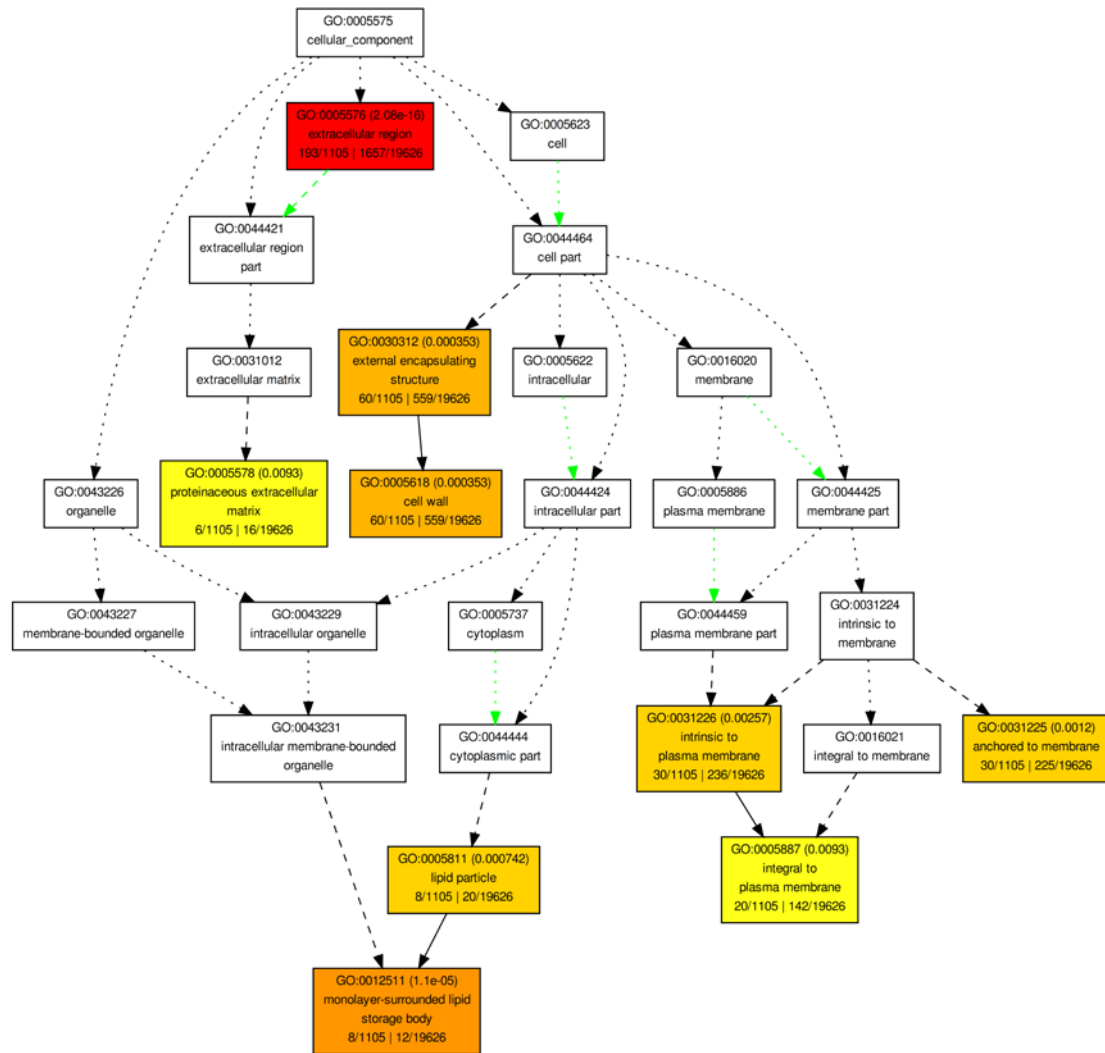

B

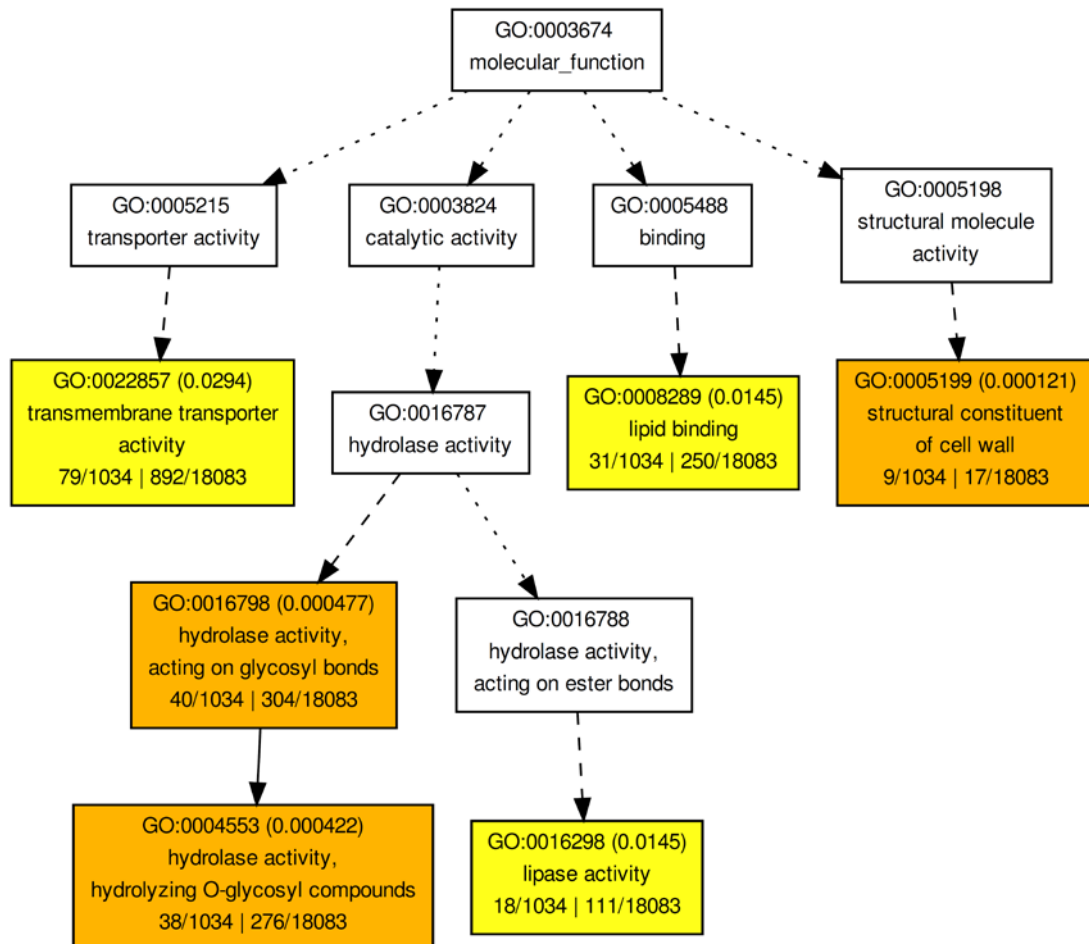

**C**

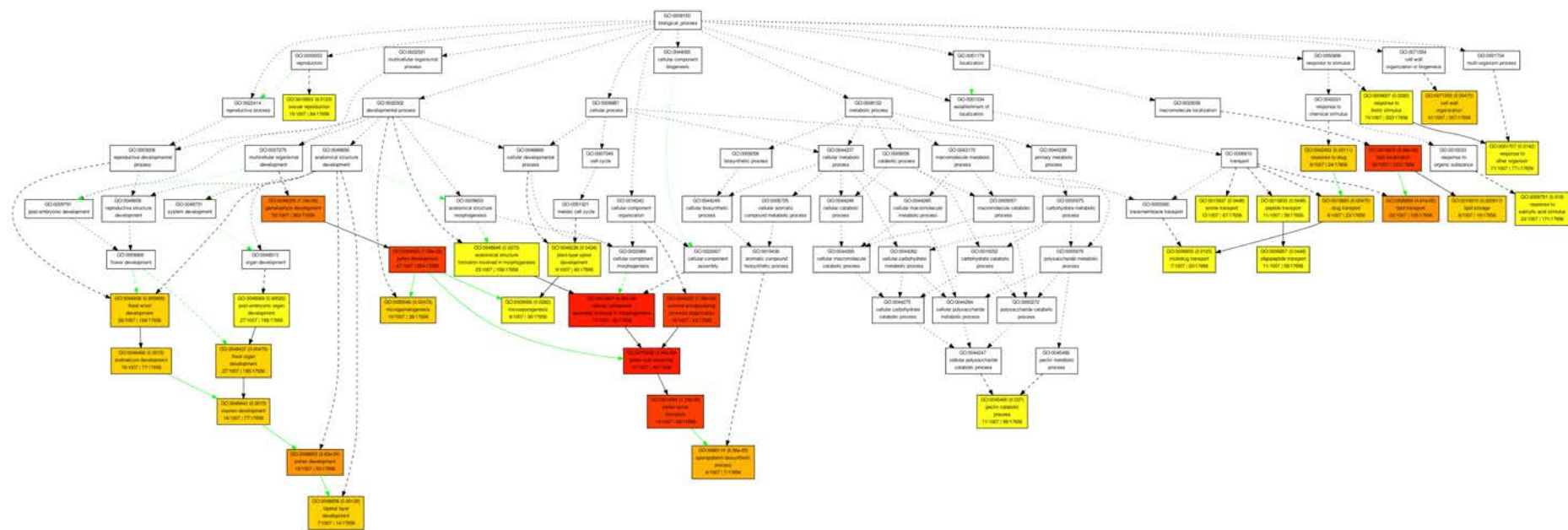

Supplement: S4 Fig — Three different graphs are shown, corresponding to terms belonging to the three main subontologies: (A) Cellular component, (B) Molecular function, and (C) Biological process. The false discovery rate (FDR) of significantly overrepresented GO terms is given in parentheses, and the corresponding graph nodes are filled in with different tones of yellow (less significant) to red (more significant). The frequency of each term in the set of differentially expressed genes and in the background set is also given, and matches the values on S2 Dataset. (PDF) [file pgen.1007182.s004.pdf]

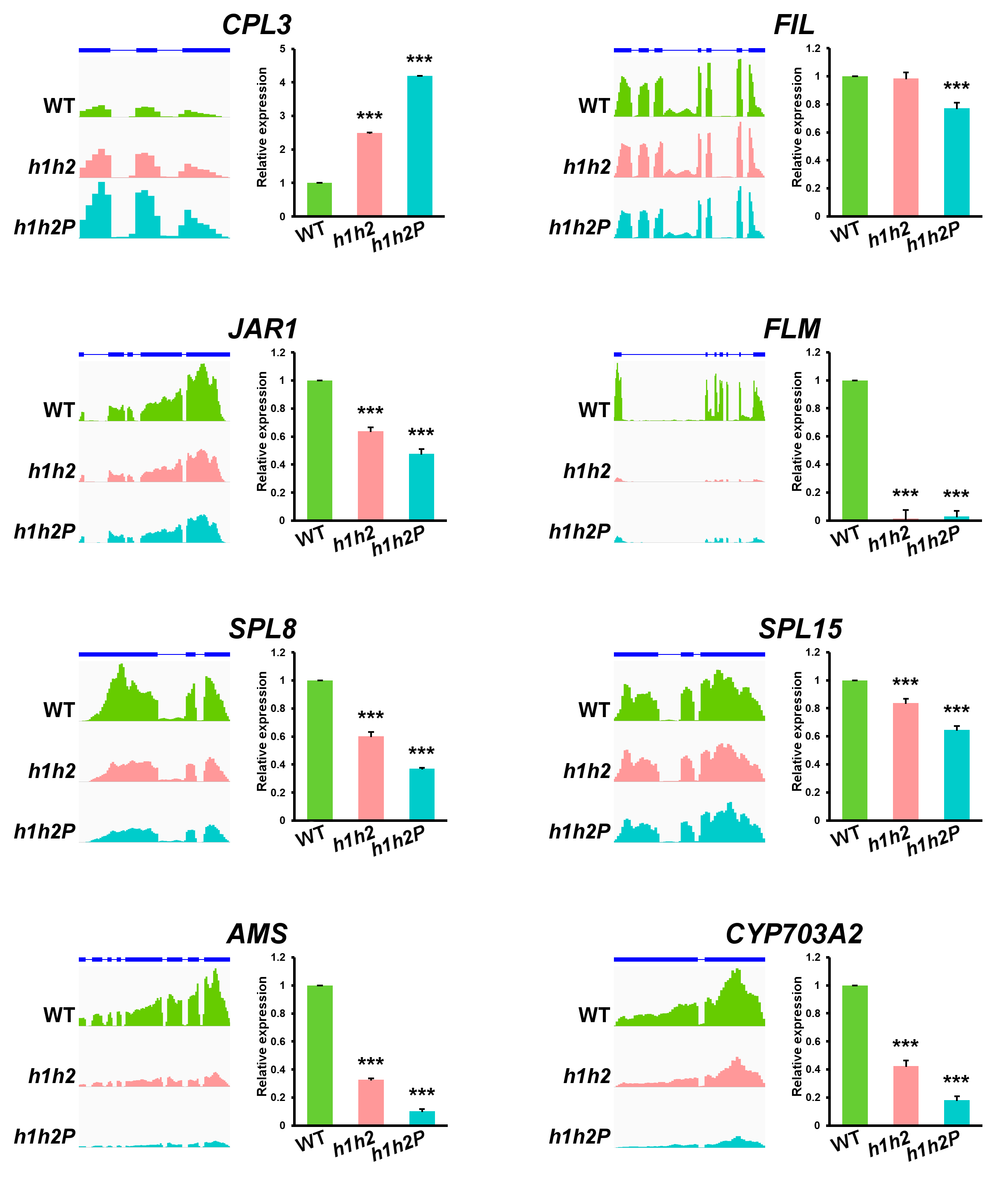

Supplement: S5 Fig — Quantification of gene expression levels of selected representative genes in Col-0 (WT) and h1h2 and h1h2P mutant backgrounds. For each gene, RNA-Seq data (normalized read counts, as determined by IGV software [108]) are shown on left panels. Annotated gene structures are depicted on the top. Thick and thin bars represent exons and introns, respectively. On right panels, monitoring of gene expression levels by qPCR is presented. Error bars denote SD. Asterisks indicate statistically significant differences with respect to WT plants (*** P < 0.001). CAPRICE-LIKE MYB3 (CPL3) is involved in trichome branching and epidermal cell differentiation [117]. The YABBY family gene FILAMENTOUS FLOWER (FIL) is involved in abaxial tissue specification and participates in flower formation [118] and the mediolateral axis of the fruit [100, 101]. JASMONATE-AMIDO SYNTHETASE 1 (JAR1) encodes the key conjugating enzyme that yields the bioactive form of jasmonate (JA), jasmonolyl-L-isoleucine (JA-Ile) [119]. FLOWERING LOCUS M (FLM) encodes a MADS-box polypeptide well known for its role as a flowering repressor, particularly in the thermosensory pathway [120,121]. SQUAMOSA PROMOTER LIKE 8 (SPL8) and SPL15 are SBP-box genes, members of the SPL family involved in various processes including flowering, stamen and sporogenesis development [122,123]. The basic helix-loop helix transcription factor-encoding ABORTED MICROSPORES (AMS) is essential for male fertility and activates the cytochrome P450 gene CYP703A2 required for sporopollenin synthesis in the anther [124]. (TIFF) [file pgen.1007182.s005.tiff]

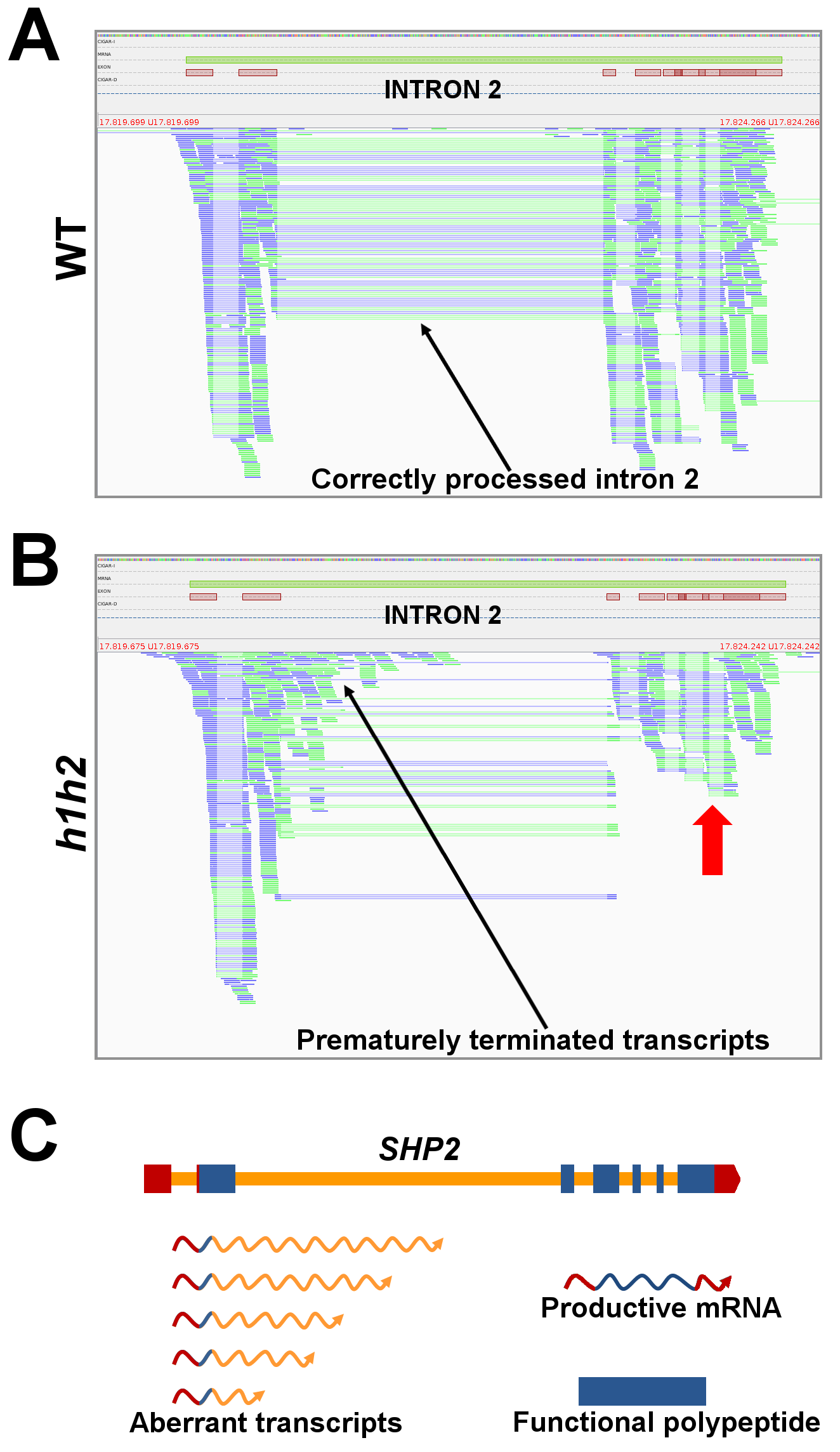

Supplement: S6 Fig — Screenshots of read coverage tracks obtained from the Tablet software [109] in Col-0 WT (A) and h1h2 (B) plants. On top of both panels, the annotated gene structure is depicted. Relative positions of exons (salmon-red bars) and intron 2 are indicated. Blue and green indicate forward and reverse reads that are properly paired according to the mapping software (Bowtie2 and Tophat). In the WT (A), abundant reads connecting exons 2 and 3 can be observed, indicative of correctly spliced exon 2. Read coverage corresponding to 3’-most exons is also abundant. In the h1h2 mutant (B) less reads corresponding to proper intron 2 splicing are detected and numerous reads corresponding to interrupted transcripts appear. In line with this, reads covering the 3’-most part of the gene decrease dramatically with respect to the wild-type (red arrow). C) Schematic representation of terminated transcripts within the SHP2 intron 2. Thick blue and red bars denote coding and non-coding exonic sequences, respectively. Thin bars represent introns. Prematurely terminated transcripts at different points inside intron 2 are represented by wavy lines partly orange in color. A solely red and blue wavy line symbolizes the fully mature mRNA encoding a functional polypeptide (thick blue bar below). (TIFF) [file pgen.1007182.s006.tiff]

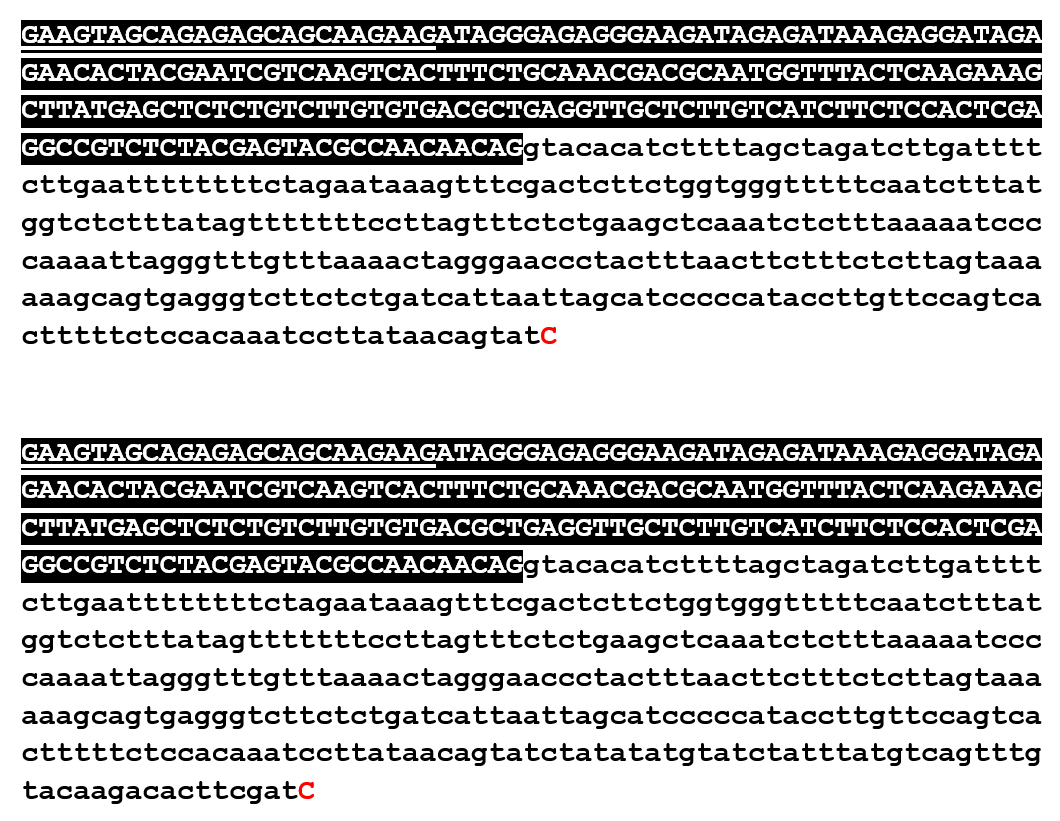

Supplement: S7 Fig — DNA sequence corresponding to exon 2 appears as white upper-case letters boxed in black. Intron 2 sequence is shown as lower-case black letters. Cleavage site is indicated (C in red). The sequence corresponding to the specific forward primer is underlined. (TIFF) [file pgen.1007182.s007.tiff]

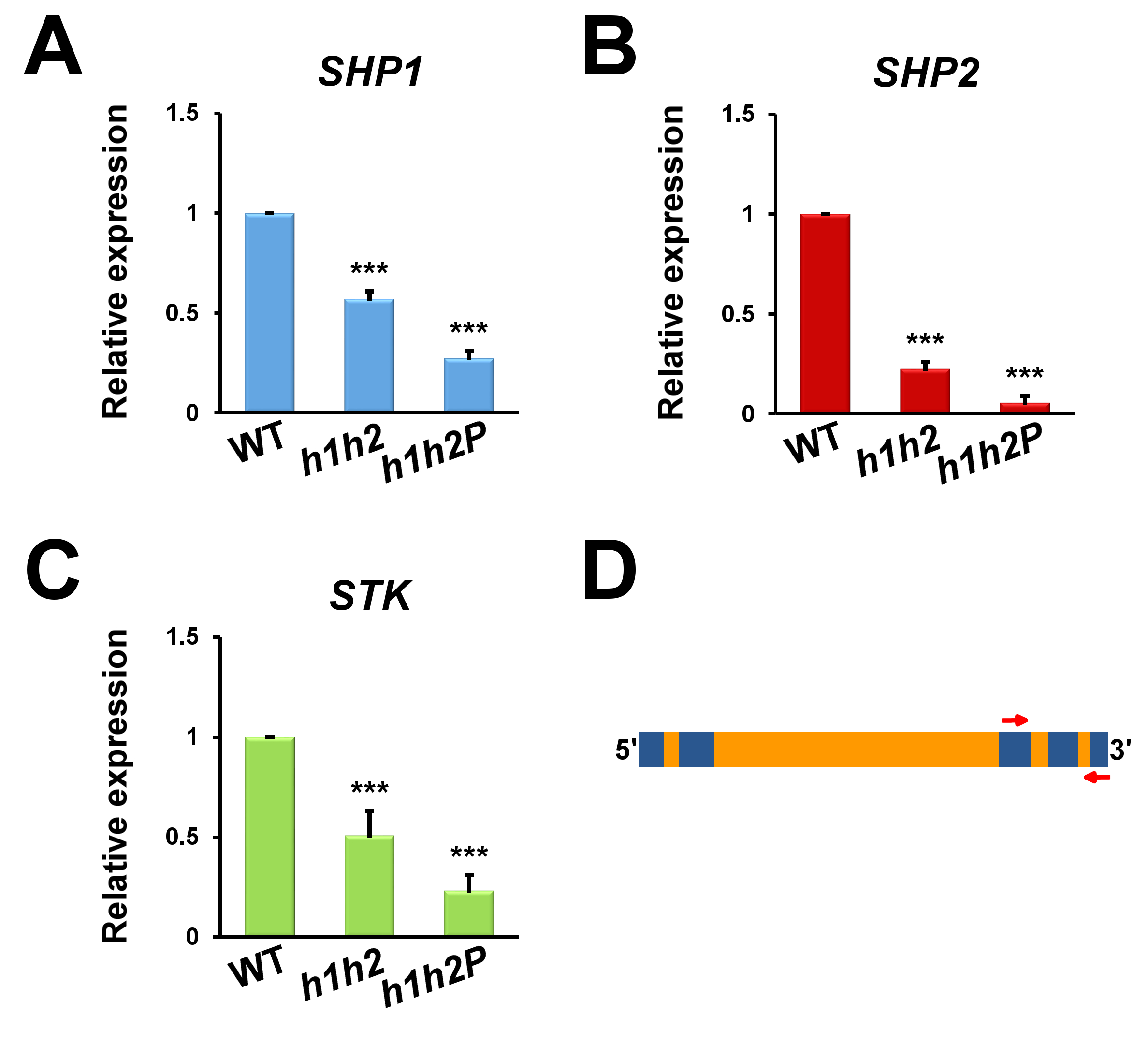

Supplement: S8 Fig — A-C) Relative expression levels, monitored by qPCR, of SHP1 (A), SHP2 (B), and STK (C) genes in wild-type plants (WT) and the h1h2 and h1h2P mutant backgrounds. Expression levels were inferred from relative abundance of correctly spliced transcripts produced at their 3’ regions, located downstream from the respective large introns. D) Schematic diagram of an idealized gene representative of the three D-class members. Blue boxes denote exons whereas intronic regions are colored in orange. Relative positions of primers used for measurements in panels A-C (red arrows) are indicated (see S2 Table for specific primer sequences). Error bars denote SD. Asterisks indicate statistically significant differences with respect to WT plants (*** P < 0.001). (TIFF) [file pgen.1007182.s008.tiff]

### At1G33080

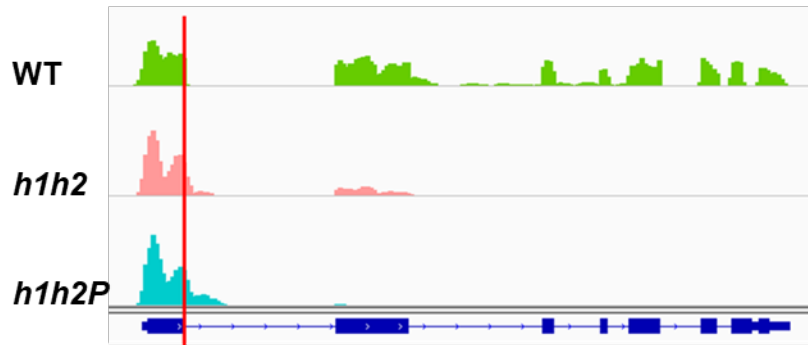

### At3G05165

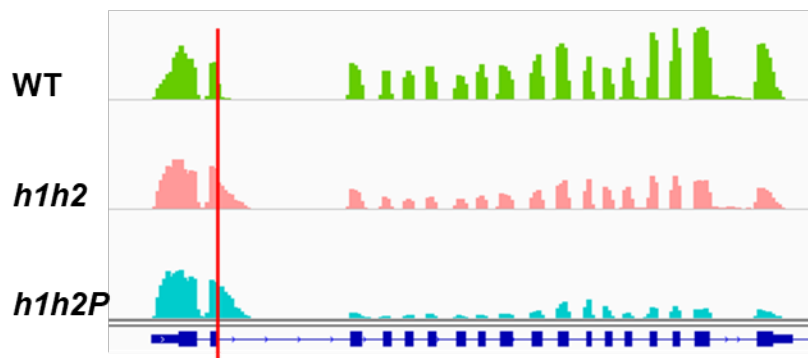

### At5G03610

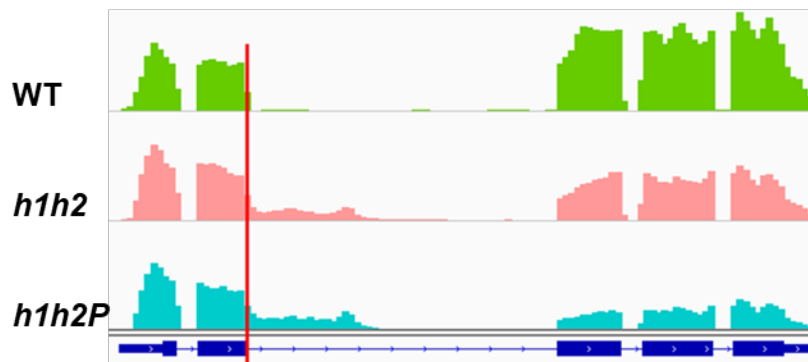

Supplement: S9 Fig — At1G33080, At3G05165 and At5G03610 transcripts abundance in Col-0 (WT), and h1h2 and h1h2P mutant backgrounds. For each gene, RNA-Seq data (normalized read counts) are shown. Annotated gene structures are depicted at the bottom of each panel. Thick and thin bars represent exons and introns, respectively. Read coverage is represented according to the IGV software [108]. Intron read areas are separated from those corresponding to preceding exons by red vertical lines. At1G33080 encodes a protein predicted as a MATE efflux family protein, an integral component of membrane with transport activity. At3G05165 encodes a major facilitator family protein, a putative integral component of membranes involved in transport. AT5G03610 encodes a GDSL-motif esterase/acyltransferase/lipase. It belongs to an enzyme group with broad substrate specificity that may catalyze acyltransfer or hydrolase reactions with lipid and non-lipid substrates. Source, TAIR (http://www.arabidopsis.org/index.jsp). (PDF) [file pgen.1007182.s009.pdf]

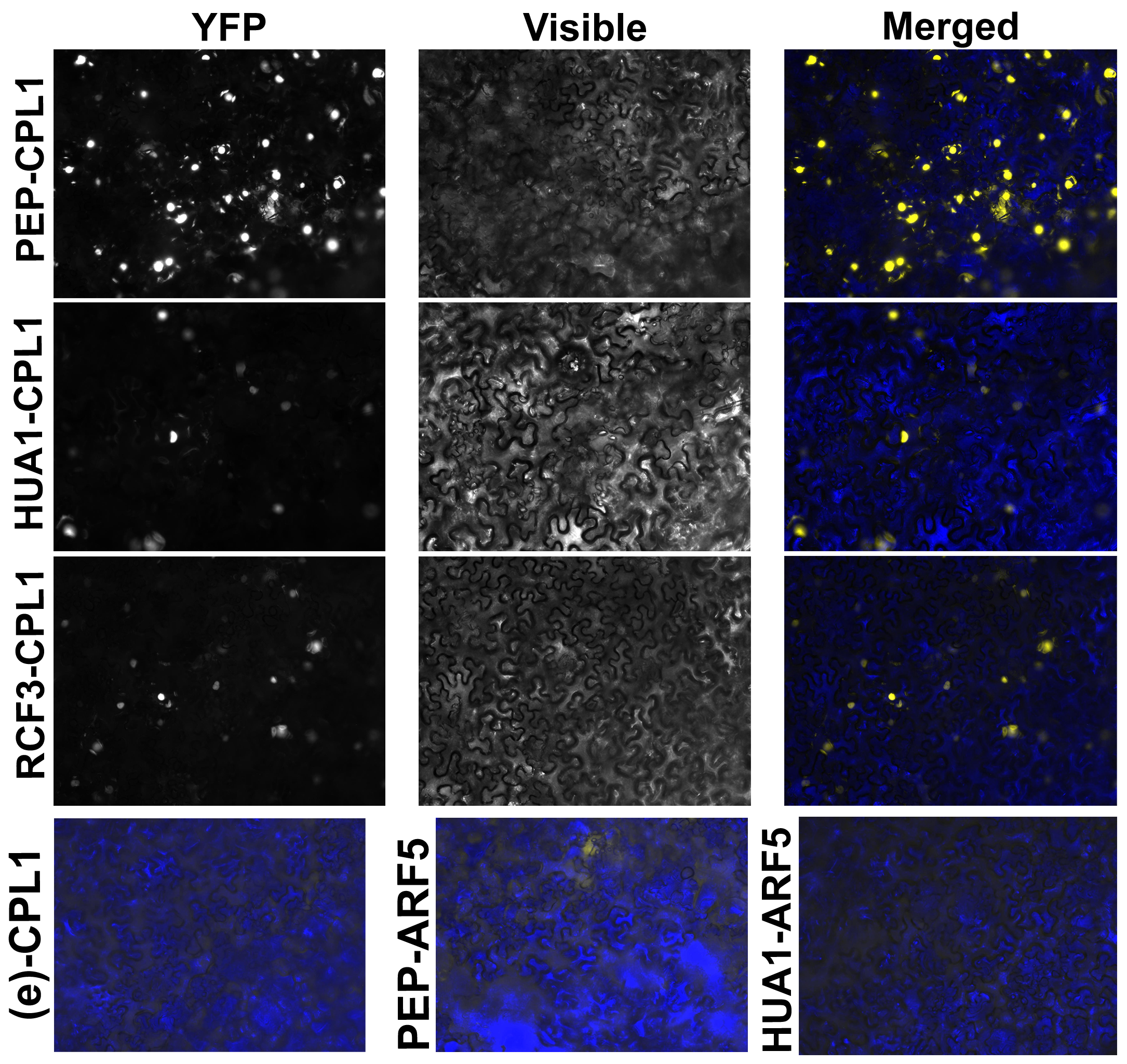

Supplement: S10 Fig — Visualization of YFP reconstitution (yellow fluorescence) in Nicotiana benthamiana leaf cells agroinfiltrated with plasmids encoding fusion proteins. The first 3 interactions show the reciprocal assays of those depicted in Fig 7. The interaction between RCF3 and CPL1 [65,66] was used as a positive control. As negative controls, Nicotiana leaves were co-infiltrated with the corresponding recombinant YFPct construct and the empty YFPnt version. The reciprocal assays were also performed and, in both cases, no signal was detected [26]. An additional control was used in which HUA1 and PEP constructs were challenged against a B3 transcription factor from the ARF5 (Auxin Response Factor 5) in both orientations, and no YFP fluorescence reconstitution was observed in these experiments. In merged visible+YFP fluorescence pictures, blue background was used to increase contrast. (TIF) [file pgen.1007182.s010.tif]
