# Supplementary material for: Ovule identity mediated by pre-mRNA processing in Arabidopsis
Source: PLoS Genet. 2018 Jan 12;14(1):e1007182. doi: 10.1371/journal.pgen.1007182 (PMC5785034; doi:10.1371/journal.pgen.1007182)
Supplement: S1 Table — The table summarizes the data of two independent rounds of counting. “Total primordia” refers to the total number of ovules produced, regardless of their viability. (PDF) [file pgen.1007182.s011.pdf]

S1 Table. Ovule abortions in *hua1-1*, *pep-4* and *hua1-1 pep-4* mutants. The table summarizes the data of two independent rounds of counting. "Total primordia" refers to the total number of ovules produced, regardless of their viability.

	Genotypes			
	**Col-0**	***pep-4***	***hua1-1***	***hua1-1 pep-4***
**Number of plants**	25	27	28	27
**Number of pistils/fruits**	100	100	100	100
**Total primordia**	6203	5649	5303	4180
**Primordia per pistil/fruit**	62,03	56,49	53,03	41,8
**Ovule abortions (total)**	121	419	509	2362
**Ovule abortions (%)**	1,94	7,51	10,12	56,58
