# Supplementary material for: Ovule identity mediated by pre-mRNA processing in Arabidopsis
Source: PLoS Genet. 2018 Jan 12;14(1):e1007182. doi: 10.1371/journal.pgen.1007182 (PMC5785034; doi:10.1371/journal.pgen.1007182)
Supplement: S2 Table — (DOCX) [file pgen.1007182.s012.docx]

**S2 Table. Oligonucleotides, genotyping and additional references.**

| **Purpose** | **Gene** | **Name** | **Oligonucleotide sequence (5’-3’)** | | |
| --- | --- | --- | --- | --- | --- |
| qPCR | *AGL18* | AGL18 f | TGTGACGCCGAGGTTGCTCT | | |
|  |  | AGL18 r | TCGCAACACAGCTTCATTTCCATG | | |
|  | *AMS* | AMS f  AMS r | GTTTTGATTCCTATACCGGGAG  GAACCCTTTTGCTGGATGTCAC | | |
|  | *CPL3* | CPL3 f  CPL3 r | TAACCATCGCAGGACTAAGCAAC  AGCGGTTCTTCCTGGGATCC | | |
|  | *CYP703A2* | CYP703 f  CYP703 r | GTTGACGTTTTGTTGTCTCTACC  CGTCGACCATTCGATTTGATC | | |
|  | *CRC* | CRC f | GCCGAACATCTCTACTACGTCC | | |
|  |  | CRC r | CACTTTTGATGCGTTGGATCTCATC | | |
|  | *FIL* | FIL f  FIL r | TCCGACCATCTCTGCTATGT  CTGTCTTTTCTCTGGAGGGC | | |
|  | *FLM* | FLM f  FLM r | GGCATAACCCTTATCGGAGATT  CAACTTTAAAAATCCAATCCGTACA | | |
|  | *INO* | INO f | TGACTGTGAGATGTGGGCAT | | |
|  |  | INO r | TTGGACTGTTCTCCTTCTCCT | | |
|  | *JAR1* | JAR1 f  JAR1 r | AACCCTGAGCTAGCTGAGACGAT  ACCACATCTCCAAGCCGGTATCGA | | |
|  | *SHP1* (E2-E6) | SHP1 ac-f | CTTGAGAACTCAGGATCAATGGAGG | | |
|  |  | SHP1 a-r | GAGGCTTCTTGCTGATAGTACTGAG | | |
|  | *SHP1* (E5-E10) | SHP1 b-f | AGCTTGTTCCGATGCCGTCAA | | |
|  |  | SHP1 b-r | ACTGTCGTCCCTTGTATCACACT | | |
|  | *SHP1* (E2-I2) | SHP1 ac-f | CTTGAGAACTCAGGATCAATGGAGG | | |
|  |  | SHP1 c-r | CCCCACTTTGTTATCATTTGAACT | | |
|  | *SHP2* (E2-E6) | SHP2 a-f | GAGGATAGAGAACACTACGAATCGTC | | |
|  |  | SHP2 a-r | AGATACATGTTATCGTTTTGCAGCTC | | |
|  | *SHP2* (E5-E7) | SHP2 b-f | CATGCAAAAAAGGGTAAAAGAAATC | | |
|  |  | SHP2 b-r | TTATACTGCCCCGACTGGTGA | | |
|  | *SHP2* (E2-I2) | SHP2 c-f | GTCTCTACGAGTACGCCAACA | | |
|  |  | SHP2 c-r | AGAAGTTAAAGTAGGGTTCCCTAG | | |
|  | *SPL8* | SPL8 f  SPL8 r | AACGACCACCGTCACATCACCA  CGGTTATGGTCAGCAAGTCGCTTAC | | |
|  | *SPL15* | SPL15 f  SPL15 r | CTTGTCATAACGAACGACGAAG  CTGGATTAATCTGCCACGGT | | |
|  | *STK* (E2-E3) | STK a-f | GGCCGTCTCTATGAATACGC | | |
|  |  | STK a-r | TGGACAGTGCTAGTGTTGGTG | | |
|  | *STK* (E3-E8) | STK b-f | AGAGGTACAAGAAAGCTTGTTCTGA | | |
|  |  | STK b-r | TGATGATGGTGTTGTTGATACCTCT | | |
|  | *STK* (E1-I1) | STK c-f | TTCTGTTTGATGCTCTTTCCCCATGA | | |
|  |  | STK c-r | ACTCCACTTTAGCTGACAAGAAAGGA | | |
|  | *SUP* | SJ1-77 | GAGATCAAACAGCATAGAGTTG | | |
|  |  | SJ1-78 | CTGTGAACATTCATGTGGCCA | | |
| **Purpose** | **Name** | **Oligonucleotide sequence (5’-3’)** | | | **PCR products size in base pairs** |
| *clf-29* genotyping | clf-29 1F | CAGCTACCTCCTCGTTCCATAGAA | | | 600 wild-type  ~700 mutant |
|  | clf-29 1R | ATGGCGTCAGAAGCTTCGCCTT | | |  |
|  | LBb1 | AACCAGCGTGGACCGCTTGCTG | | |  |
| *hen4-2* genotyping | hen4-2 f  hen4-2 r | AGAGAAGCCATTTTCCATATTACAAG**CACGCTG**  TCCTCAGATCTTCTGTGCAGACTTGAG | | | 206 wild-type  176+30 mutant (DraIII) |
| The hen4-2 f oligonucleotide introduces a point mutation (grey-shaded C) to create a DraIII site (bold-type) in the mutant | | | | | |
| **Purpouse** | **Name** | **Oligonucleotide sequence (5’-3’)** | | **Modifications** | |
|  | bifc ARF5-f | CTATTTACAATTGTCGACATGATGGCTTCATTGTCTTGTG | | Ref [115] | |
|  | bifc ARF5-r | TTTCGAACCCGGGGTACCTGAAACAGAAGTCTTAAGATCG | | Ref [115] | |
| BiFC | bifc CPL1-f | CTGCAGACGCGTCTCGAG**GAATTC**ATGTATAGTAATAATAGAGTAGAAGTG | | DNA assembly tail (EcoRI) | |
|  | bifc CPL1-r | TTTCGAACCCGGGGTACC**GAATTC**AGAGTATCTTCCCGAAGATGGCATCCG | | DNA assembly tail (EcoRI) | |
|  | bifc HUA1-f | CTATTTACAATT**GTCGAC**ATGGCACATCGTCAATTGTATAG | | DNA assembly tail (SalI) | |
|  | bifc HUA1-r | TTTCGAACCCGG**GGTACC**TTGAGTAGTGTCGGTGTTGGTTGC | | DNA assembly tail (KpnI) | |
|  | bifc PEP-f | CTATTTACAATT**GTCGAC**TGGCCGCCGTCGCAGATTCCGTTG | | DNA assembly tail (SalI) | |
|  | bifc PEP-R | TTTCGAACCCGG**GGTACC**AAGATTATAACTGCTGTAGCCACC | | DNA assembly tail (KpnI) | |
|  | bifc RCF3-f | CTATTTACAATT**GTCGAC**ATGGAGAGATCTAGATCC | | DNA assembly tail (SalI) | |
|  | bifc RCF3-r | TTTCGAACCCGG**GGTACC**CGGTCCATCCTCTTGTATG | | DNA assembly tail (KpnI) | |
|  |  |  | |  | |
|  |  |  | |  | |
| Y2H | pGILDA-CPL1-f | AACGGCGACTGGCTG**GAATTC**ATGTATAGTAATAATAGAGTAGAAGTG | | DNA assembly tail (EcoRI) | |
|  | pGILDA-CPL1-r | TTGGCTGCAGGTCGA**CTCGAG**TTAAGAGTATCTTCCCGAAGATGGCATCCG | | DNA assembly tail (XhoI) | |
|  | pB42AD-CPL1-f | GATTATGCCTCTCCC**GAATTC**ATGTATAGTAATAATAGAGTAGAAGTG | | DNA assembly tail (EcoRI) | |
|  | pB42AD-CPL1-r | AGAAGTCCAAAGCTT**CTCGAG**TTAAGAGTATCTTCCCGAAGATGGCATCCG | | DNA assembly tail (XhoI) | |
|  | pGILDA-HUA1-f | AACGGCGACTGGCTG**GAATTC**ATGGCACATCGTCAATTGTATAG | | DNA assembly tail (EcoRI) | |
|  | pGILDA-HUA1-r | TTGGCTGCAGGTCGA**CTCGAG**TCATTGAGTAGTGTCGGTG | | DNA assembly tail (XhoI) | |
|  | pB42AD-HUA1-f | GATTATGCCTCTCCC**GAATTC**ATGGCACATCGTCAATTGTATAG | | DNA assembly tail (EcoRI) | |
|  | pB42AD-HUA1-r | AGAAGTCCAAAGCTT**CTCGAG**TCATTGAGTAGTGTCGGTG | | DNA assembly tail (XhoI) | |
|  | pGILDA-PEP-f | AACGGCGACTGGCTG**GAATTC**ATGGCCGCCGTCGCAGATTCCG | | DNA assembly tail (EcoRI) | |
|  | pGILDA-PEP-r | TTGGCTGCAGGTCGA**CTCGAG**TCAAAGATTATAACTGCTGTAG | | DNA assembly tail (XhoI) | |
|  | pB42AD-PEP-f | GATTATGCCTCTCCC**GAATTC**ATGGCCGCCGTCGCAGATTCCG | | DNA assembly tail (EcoRI) | |
|  | pB42AD-PEP-r | AGAAGTCCAAAGCTT**CTCGAG**TCAAAGATTATAACTGCTGTAG | | DNA assembly tail (XhoI) | |
|  | pGILDA-RCF3-f | AACGGCGACTGGCTG**GAATTC**ATGGAGAGATCTAGATCC | | DNA assembly tail (EcoRI) | |
|  | pGILDA-RCF3-r | TTGGCTGCAGGTCGA**CTCGAG**TCACGGTCCATCCTCTTGTATGC | | DNA assembly tail (XhoI) | |
|  | pB42AD-RCF3-f | GATTATGCCTCTCCC**GAATTC**ATGGAGAGATCTAGATCC | | DNA assembly tail (EcoRI) | |
|  | pB42AD-RCF3-r | AGAAGTCCAAAGCTT**CTCGAG**TCACGGTCCATCCTCTTGTATGC | | DNA assembly tail (XhoI) | |

Sequences for DNA assembly are marked in grey. Bold-type letters indicate restriction sites.

In qPCR experiments, E and I (with numerals, between brackets) refer to exons and introns, respectively.

The rest of genotyping procedures were as previously reported:

*flk-2* [47]

*hua1-1* (Western et al., 2002)

*hua2-1* (Western et al., 2002)

*hua2-4* [47]

*hua2-7* [26]

*pep-4* [43]

*35S::PEP* [26]

**Supplementary reference**

Western TL, Cheng Y, Liu J, Chen X. HUA ENHANCER2, a putative DExH-box RNA helicase, maintains homeotic B and C gene expression in Arabidopsis. Development. 2002;129: 1569–81.
